# Risk factors for death, stroke, and bleeding in 28,628 patients from the GARFIELD-AF registry: Rationale for comprehensive management of atrial fibrillation

**DOI:** 10.1371/journal.pone.0191592

**Published:** 2018-01-25

**Authors:** Jean-Pierre Bassand, Gabriele Accetta, Wael Al Mahmeed, Ramon Corbalan, John Eikelboom, David A. Fitzmaurice, Keith A. A. Fox, Haiyan Gao, Samuel Z. Goldhaber, Shinya Goto, Sylvia Haas, Gloria Kayani, Karen Pieper, Alexander G. G. Turpie, Martin van Eickels, Freek W. A. Verheugt, Ajay K. Kakkar, for the GARFIELD-AF Investigators

**Affiliations:** 1 Department of Cardiology–EA 3920, University of Besançon, Besançon, France; 2 Thrombosis Research Institute, London, United Kingdom; 3 Cardiology, Heart and Vascular Institute, Cleveland Clinic Abu Dhabi, Abu Dhabi, United Arab Emirates; 4 Department of Cardiology, Catholic University School of Medicine, Santiago, Chile; 5 Department of Medicine, McMaster University, Hamilton, Canada; 6 Warwick Medical School, University of Warwick, Coventry, United Kingdom; 7 Centre for Cardiovascular Science, University of Edinburgh, Edinburgh, United Kingdom; 8 Division of Cardiovascular Medicine, Brigham and Women’s Hospital and Harvard Medical School, Boston, Massachusetts, United States of America; 9 Department of Medicine (Cardiology), Tokai University School of Medicine, Kanagawa, Japan; 10 Formerly Klinikum rechts der Isar, Technical University of Munich, Munich, Germany; 11 Duke Clinical Research Institute, Durham, North Carolina, United States of America; 12 Bayer AG, Berlin, Germany; 13 Department of Cardiology, Onze Lieve Vrouwe Gasthuis, Amsterdam, The Netherlands; 14 University College London, London, United Kingdom; Klinikum Region Hannover GmbH, GERMANY

## Abstract

**Background:**

The factors influencing three major outcomes–death, stroke/systemic embolism (SE), and major bleeding–have not been investigated in a large international cohort of unselected patients with newly diagnosed atrial fibrillation (AF).

**Methods and results:**

In 28,628 patients prospectively enrolled in the GARFIELD-AF registry with 2-year follow-up, we aimed at analysing: (1) the variables influencing outcomes; (2) the extent of implementation of guideline-recommended therapies in comorbidities that strongly affect outcomes. Median (IQR) age was 71.0 (63.0 to 78.0) years, 44.4% of patients were female, median (IQR) CHA_2_DS_2_-VASc score was 3.0 (2.0 to 4.0); 63.3% of patients were on anticoagulants (ACs) with or without antiplatelet (AP) therapy, 24.5% AP monotherapy, and 12.2% no antithrombotic therapy. At 2 years, rates (95% CI) of death, stroke/SE, and major bleeding were 3.84 (3.68; 4.02), 1.27 (1.18; 1.38), and 0.71 (0.64; 0.79) per 100 person-years. Age, history of stroke/SE, vascular disease (VascD), and chronic kidney disease (CKD) were associated with the risks of all three outcomes. Congestive heart failure (CHF) was associated with the risks of death and stroke/SE. Smoking, non-paroxysmal forms of AF, and history of bleeding were associated with the risk of death, female sex and heavy drinking with the risk of stroke/SE. Asian race was associated with lower risks of death and major bleeding versus other races. AC treatment was associated with 30% and 28% lower risks of death and stroke/SE, respectively, compared with no AC treatment. Rates of prescription of guideline-recommended drugs were suboptimal in patients with CHF, VascD, or CKD.

**Conclusions:**

Our data show that several variables are associated with the risk of one or more outcomes, in terms of death, stroke/SE, and major bleeding. Comprehensive management of AF should encompass, besides anticoagulation, improved implementation of guideline-recommended therapies for comorbidities strongly associated with outcomes, namely CHF, VascD, and CKD.

**Trial registration:**

ClinicalTrials.gov NCT01090362

## Introduction

Atrial fibrillation (AF), the most frequent of all sustained cardiac arrhythmias, is associated with increased risk of death, stroke/systemic embolism (SE), and bleeding. Currently recommended management approaches include rhythm and/or rate control, and anticoagulation for the prevention of stroke/SE in at-risk patients without contraindication [1, 2]. We previously showed in the Global Anticoagulant Registry in the FIELD–Atrial Fibrillation (GARFIELD-AF) registry that at 2-year follow-up, death was the most frequent major adverse event, occurring at a much higher rate than stroke/SE or major bleeding [3]. Stroke-related death accounted for less than 10% of all causes of death.

In this report, we analyse at 2-year follow-up the outcomes of 28,628 patients with newly diagnosed AF recruited in the first three cohorts of GARFIELD-AF, with two objectives. The primary objective was to identify the variables associated with the risks of all three major outcome measures, namely death, stroke/SE and bleeding, particularly those linked to modifiable risk factors. The secondary objective was to assess compliance with guidelines as regards drug prescription in comorbidities identified to strongly affect outcomes, namely congestive heart failure (CHF), vascular disease (VascD), and chronic kidney disease (CKD) [4–6].

## Methods

The design of the GARFIELD-AF registry was reported previously [7, 8]. Briefly, men and women aged ≥18 years with non-valvular AF diagnosed according to standard local procedures within the previous 6 weeks, and with at least one non-prespecified risk factor for stroke as judged by the investigator, were eligible for inclusion [8].

Patients were enrolled prospectively and consecutively. Investigator sites were selected randomly (apart from 18 sites) and represent the different care settings in each participating country (office-based practice; hospital departments including neurology, cardiology, geriatrics, internal medicine and emergency; anticoagulation clinics; and general or family practice) [7, 8].

### Ethics statement

Independent ethics committee and hospital-based institutional review board approvals were obtained. A list of central ethics committees and regulatory authorities that provided approval can be found in S2 File. Additional approvals were obtained from individual study sites. The registry is being conducted in accordance with the principles of the Declaration of Helsinki, local regulatory requirements, and the International Conference on Harmonisation–Good Pharmacoepidemiological and Clinical Practice guidelines. Written informed consent is obtained from all study participants. Confidentiality and anonymity of all patients recruited into this registry are maintained.

### Procedures and outcome measures

Baseline characteristics collected at inclusion in the registry included medical history, care setting, type of AF, date and method of diagnosis, symptoms, antithrombotic treatment (vitamin K antagonists [VKAs], non-vitamin K antagonist oral anticoagulants [NOACs], and antiplatelet [AP] treatment), as well as all cardiovascular drugs. Race was classified by the investigator in agreement with the patient [8]. Data on components of the CHA_2_DS_2_-VASc and HAS-BLED risk stratification schemes were collected to assess the risks of stroke and bleeding retrospectively. HAS-BLED scores were calculated excluding fluctuations in international normalised ratio.

Collection of follow-up data occurred at 4-monthly intervals up to 24 months [7, 8]. Standardised definitions for clinical events have been reported previously [7, 8]. In brief, baseline characteristics and treatments, and the incidence of death (cardiovascular and non-cardiovascular), stroke/SE, and bleeding were recorded. Submitted data were examined for completeness and accuracy by the coordinating centre (Thrombosis Research Institute, London, UK), and data queries were sent to study sites. GARFIELD-AF data are captured using an electronic case report form (eCRF). In accordance with the study protocol, 20% of all eCRFs are monitored against source documentation [9].

Data for the analysis in this report were extracted from the study database on 28 July 2016.

### Definitions

VascD included peripheral artery disease or coronary artery disease with a history of acute coronary syndromes (ACS). CKD was classified according to National Kidney Foundation guidelines into two groups: moderate-to-severe (stages 3–5), or mild (stages 1 and 2) or none [6]. CHF was defined as current/prior history of CHF or left ventricular ejection fraction of <40%.

Guideline-recommended therapies included angiotensin-converting enzyme (ACE) inhibitors/angiotensin receptor blockers (ARBs), betablockers, aldosterone blockade, and loop diuretics for CHF; aspirin (ASA), statins, ACE inhibitors/ARBs, and betablockers for VascD; and ACE inhibitors/ARBs for CKD.

### Statistical analysis

Continuous variables are expressed as median (interquartile range, IQR) and categorical variables as frequency and percentage. Baseline rates of prescription of guideline-recommended drugs in CHF, VascD, and CKD are displayed with the baseline rate of prescription of anticoagulants (ACs).

Differences in medians were tested using the Wilcoxon rank-sum (Mann-Whitney) test. Differences in proportions were tested using the two-sample test of proportion. Trends were assessed using an extension of the Wilcoxon rank-sum test.

Occurrence of major clinical outcomes is expressed as person-time event rates (per 100 person-years) and 95% confidence intervals (CIs). Person-year rates were estimated using a Poisson model with the number of events as the dependent variable and the log of time as an offset, i.e., a covariate with a known coefficient of 1. Only the first occurrence of events was taken into account. Hazard ratios (HRs) and 95% CIs were estimated using a proportional hazards Cox model based on five imputed datasets. The imputed datasets were created by the Multiple Imputation by Chained Equations (MICE) algorithm [10, 11]. The proportional hazard assumption was assessed visually using plots of the survivor function. The following variables were included in the Cox model: age groups (<65, 65–69, 70–74, ≥75 years), gender, race (Caucasian/Hispanic/Latino, Asian, other race–including Afro-Caribbean, mixed/other, and unwilling to declare/not recorded), smoking (no, ex, current), diabetes, hypertension, previous stroke/transient ischaemic attack (TIA)/SE, history of bleeding, cardiac failure, VascD, moderate-to-severe renal disease, AC treatment, type of AF (new onset [unclassified], paroxysmal, persistent, permanent), and alcohol consumption (abstinent, light, moderate, heavy). Hazard ratios were adjusted for all variables in the model.

Data analysis was performed with SAS version 9.4 (SAS Institute Inc., Cary, NC, USA) and Stata Statistical Software: Release 14.2 (StataCorp, College Station, TX, USA). Forest plots were created in R 3.3.1 (R Core Team, Vienna, Austria).

## Results

### Study population

The study population comprised 28,628 prospective patients with AF enrolled in GARFIELD-AF between March 2010 and October 2014 and followed for 2 years. Patients came from 1048 study sites representative of routine practice in each of 32 countries. Two-year follow-up was achieved in 91% of patients. At baseline, the median (IQR) age was 71.0 (63.0 to 78.0) years and 44.4% of patients were female. The median (IQR) CHA_2_DS_2_-VASc and HAS-BLED scores were 3.0 (2.0 to 4.0) and 1.0 (1.0 to 2.0), respectively. Other baseline characteristics are shown in Table 1. At diagnosis of AF, 63.3% of patients were prescribed AC therapy (46.3% VKAs and 17.0% NOACs, with or without APs), 24.5% received AP monotherapy, and 12.2% received no AC or AP therapy.

**Table 1:** Baseline characteristics of all patients (N = 28,628).

Variable	Value	%
Female, n/n, %	12,711/28,628	44.4
Age, median (IQR), years	71.0 (63.0 to 78.0)	n/a
**Age group, n/n, %**		
<65 years	8517/28,628	29.8
65–74 years	9323/28,628	32.6
≥75 years	10,788/28,628	37.7
**Race, n/n, %**		
Afro-Caribbean	53/28,628	0.2
Asian (not Chinese)	5621/28,628	19.6
Chinese	1541/28,628	5.4
Caucasian	18,199/28,628	63.6
Hispanic/Latino	2032/28,628	7.1
Mixed/other	407/28,628	1.4
Unwilling to declare/not recorded	775/28,628	2.7
Body mass index, median (IQR), kg/m^2^	27.0 (24.0 to 31.0)	n/a
Pulse, median (IQR), bpm	84.0 (70.0 to 105.0)	n/a
Systolic blood pressure, median (IQR), mm Hg	131.0 (120.0 to 145.0)	n/a
Diastolic blood pressure, median (IQR), mm Hg	80.0 (70.0 to 89.0)	n/a
Left ventricular ejection fraction <40%, n/n (%)	1665/16,379	10.2
**Type of atrial fibrillation, n/n (%)**		
Permanent	3638/28,626	12.7
Persistent	4367/28,626	15.3
Paroxysmal	7681/28,626	26.8
New onset (unclassified)	12,940/28,626	45.2
**Medical history, n/n (%)**		
Congestive heart failure	3532/17,158	20.6
Coronary artery disease	3418/17,158	19.9
Acute coronary syndromes	1613/17,155	9.4
Carotid occlusive disease	867/28,425	3.1
Pulmonary embolism/deep vein thrombosis	768/28,535	2.7
Coronary artery bypass graft	854/28,067	3.0
Stroke/transient ischaemic attack	3424/28,626	12.0
Systemic embolism	194/28,537	0.7
History of bleeding	780/28,553	2.7
History of hypertension	22,161/28,583	77.5
Hypercholesterolaemia	11,514/28,080	41.0
Diabetes mellitus	6211/28,626	21.7
Cirrhosis	154/28,350	0.5
Chronic renal disease, n/n (%)		
None or mild (Grades I and II)	25,655/28,625	89.6
Moderate to severe (Grades III to V)	2970/28,625	10.4
Dementia	410/28,514	1.4
**Alcohol consumption, n/n (%)**		
Abstinent/light	21,278/24,218	87.9
Moderate	2335/24,218	9.6
Heavy	605/24,218	2.5
Current/previous smoker, n/n (%)	9062/26,046	34.8
**Antithrombotic treatment, n/n (%)**		
Vitamin K antagonists	9947/28,221	35.2
Vitamin K antagonists + antiplatelet	3127/28,221	11.1
Factor Xa inhibitors	2300/28,221	8.1
Factor Xa inhibitors + antiplatelet	643/28,221	2.3
Direct thrombin inhibitors	1499/28,221	5.3
Direct thrombin inhibitors + antiplatelet	356/28,221	1.3
Antiplatelet only	6905/28,221	24.5
None	3444/28,221	12.2
CHA_2_DS_2_-VASc score, median (IQR)	3.0 (2.0 to 4.0)	n/a
**CHA**_**2**_**DS**_**2**_**-VASc score categories, n/n (%)**		
0	687/27,973	2.5
1	3352/27,973	12.0
2	5539/27,973	19.8
3	6753/27,973	24.1
4	6165/27,973	22.0
5	3255/27,973	11.6
6–9	2222/27,973	7.9
HAS-BLED score, median (IQR)*	1.5 (0.9)	n/a
HAS-BLED score categories, n/n (%)*		
0	2494/19,402	12.9
1	7998/19,402	41.2
2	6483/19,402	33.4
3	2054/19,402	10.6
4	338/19,402	1.7
5	34/19,402	0.2
6–9	1/19,402	0.0
**Care setting specialty at diagnosis, n/n (%)**		
Cardiology	18,217/28,626	63.6
Geriatrics	117/28,626	0.4
Internal medicine	5287/28,626	18.5
Neurology	568/28,626	2.0
Primary care/general practice	4437/28,626	15.5

CHA_2_DS_2_-VASc, cardiac failure, hypertension, age ≥75 (doubled), diabetes, stroke (doubled)-vascular disease, age 65–74, and sex category (female); IQR, interquartile range.

*‘modified’ HAS-BLED, hypertension, abnormal renal/liver function (1 point each), stroke, bleeding history or predisposition, elderly (>65), drugs/alcohol concomitantly (1 point each).

### Baseline therapies

In patients with CHF, the rates of prescription of guideline-recommended therapies were no drug in 11.7%, one drug in 25.1%, two drugs in 33.6%, three drugs in 23.3%, and four drugs in 6.4% of patients (Table 2). These figures were not affected by the heart failure stage (New York Heart Association I–II vs III–IV).

**Table 2:** Baseline prescription of guideline-recommended therapies in patients with congestive heart failure or vascular disease*.

Number of guideline-recommended drugs, n (%)	Patients with congestive heart failure	Patients with vascular disease
0	781 (11.7)	248 (6.0)
1	1675 (25.1)	708 (17.0)
2	2243 (33.6)	1205 (28.9)
3	1556 (23.3)	1320 (31.7)
4	424 (6.4)	685 (16.4)
Total	6679 (100)	4166 (100)

*Guideline-recommended therapies for congestive heart failure: angiotensin-converting enzyme (ACE) inhibitors/angiotensin receptor blockers (ARBs), betablockers, aldosterone blockade, and loop diuretics; guideline-recommended therapies for vascular disease: aspirin, statins, ACE inhibitors/ARBs, and betablockers.

In patients with VascD, the rates of prescription of guideline-recommended therapies were no drug in 6.0%, one drug in 17.0%, two drugs in 28.9%, three drugs in 31.7%, and four drugs in 16.4% (Table 2).

Details about guideline-recommended drugs for both CHF and VascD are in Table 3.

**Table 3:** Monotherapy and combinations of guideline-recommended therapies prescribed for patients with congestive heart failure or vascular disease.

Guideline-recommended therapies	n (%)
**Congestive heart failure**	
1 drug	
ACE inhibitor/ARB	757 (12.8)
BB	441 (7.5)
Aldosterone blockade	51 (0.9)
Loop diuretic	426 (7.2)
2 drugs	
ACE inhibitor/ARB + BB	707 (12.0)
ACE inhibitor/ARB + aldosterone blockade	106 (1.8)
ACE inhibitor/ARB + loop diuretic	976 (16.5)
BB + aldosterone blockade	33 (0.6)
BB + loop diuretic	318 (5.4)
Aldosterone blockade + loop diuretic	103 (1.8)
3 drugs	
ACE inhibitor/ARB + BB + aldosterone blockade	119 (2.0)
ACE inhibitor/ARB + BB + loop diuretic	1046 (17.7)
ACE inhibitor/ARB + aldosterone blockade + loop diuretic	295 (5.0)
BB + aldosterone blockade + loop diuretic	96 (1.6)
4 drugs	
ACE inhibitor/ARB + BB + aldosterone blockade + loop diuretic	424 (7.2)
**Vascular disease**	
1 drug	
ASA	123 (3.0)
BB	116 (3.0)
Statin	197 (5.0)
ACE inhibitor/ARB	272 (6.9)
2 drugs	
ASA + BB	91 (2.3)
ASA + statin	261 (6.7)
ASA + ACE inhibitor/ARB	175 (4.5)
BB + statin	132 (3.4)
BB + ACE inhibitor/ARB	166 (4.2)
Statin + ACE inhibitor/ARB	380 (9.7)
3 drugs	
ASA + BB + statin	234 (6.0)
ASA + BB + ACE inhibitor/ARB	135 (3.4)
ASA + statin + ACE inhibitor/ARB	521 (13.3)
BB + statin + ACE inhibitor/ARB	430 (11.0)
4 drugs	
ASA + BB + statin + ACE inhibitor/ARB	685 (17.5)

ACE, angiotensin-converting enzyme; ARB, angiotensin receptor blocker; ASA, aspirin; BB, betablocker.

Of the patients with CKD, 36.1% were prescribed ACE inhibitors/ARBs, drugs that may slow the rate of kidney function deterioration.

### Clinical outcomes

During 2-year follow-up, the rates (95% CI) of all-cause mortality, stroke/SE, and major bleeding (first occurrences) were 3.84 (3.68; 4.02), 1.27 (1.18; 1.38), and 0.71 (0.64, 0.79) per 100 person-years, respectively (Table 4). Cardiovascular death occurred at a rate of 1.46 (1.36; 1.57) per 100 person-years and constituted 37.9% of deaths (Tables 4 and 5). Non-cardiovascular death occurred at a rate of 1.44 (1.34; 1.55) per 100-person-years and represented 37.4% of deaths (Tables 4 and 5). The most frequent known causes of death were CHF (12.1% of all deaths) and malignancy (11.1% of all deaths) (Table 5).

**Table 4:** Two-year event rates including additional outcomes.

Outcome	N	Events	%	Event rate (per 100 person-years)	95% CI
All-cause death	28,628	1985	6.9	3.84	3.68; 4.02
Cardiovascular death	28,628	753	2.6	1.46	1.36; 1.57
Non-cardiovascular death	28,628	743	2.6	1.44	1.34; 1.55
Undetermined cause of death	28,628	489	1.7	0.95	0.87; 1.04
Stroke/SE	28,628	651	2.3	1.27	1.18; 1.38
Major bleed	28,628	366	1.3	0.71	0.64; 0.79
MI/ACS	28,628	348	1.2	0.68	0.61; 0.75
Congestive heart failure	28,628	988	3.5	1.96	1.84; 2.08

ACS, acute coronary syndromes; CI, confidence interval; MI, myocardial infarction; SE, systemic embolism.

**Table 5:** Breakdown of primary outcomes by type of event at 2-year follow-up.

Event	n (%)
**All-cause death**	1985
Cardiovascular causes	753 (37.9)
*Congestive heart failure*	240 (12.1)
*Sudden/unwitnessed death*	129 (6.5)
*Acute coronary syndromes*	82 (4.1)
*Ischaemic stroke*	88 (4.4)
*Other*	214 (10.8)
Non-cardiovascular causes	743 (37.4)
*Malignancy*	220 (11.1)
*Respiratory failure*	150 (7.6)
*Infection/sepsis*	147 (7.4)
*Renal*	49 (2.5)
*Suicide*	1 (0.1)
*Other*	176 (8.9)
Undetermined causes	489 (24.6)
Stroke (not including systemic embolism)*	625
Primary ischaemic stroke	441 (70.6)
*Of which secondary haemorrhagic ischaemic stroke*	27 (4.3)
Primary intracerebral haemorrhage	66 (10.6)
*Intracerebral*	41 (6.6)
*Subarachnoid*	5 (0.8)
*Intraventricular*	7 (1.1)
*Unknown*	13 (2.1)
Undetermined	118 (18.9)
Bleeding events (not including minor bleeds)*	866
Severity of bleed	
*Non-major clinically relevant*	500 (57.7)
*Major*	366 (42.3)
Fatal	60 (6.9)

*Only the first occurrence of each event was taken into account.

Of the strokes occurring during follow-up, 70.6% were primary ischaemic, 10.6% were primary intracerebral haemorrhages, and 18.9% were undetermined (Table 5). Bleeding events were more likely to be non-major clinically relevant (57.7%) than major (42.3%), and a minority were fatal (6.9%; Table 5).

The rates (95% CI) of myocardial infarction/ACS and of new onset or worsening of CHF during follow-up were 0.68 (0.61; 0.75) and 1.96 (1.84; 2.08) per 100 person-years, respectively (Table 4).

### Factors influencing outcomes

**Fig 1:**
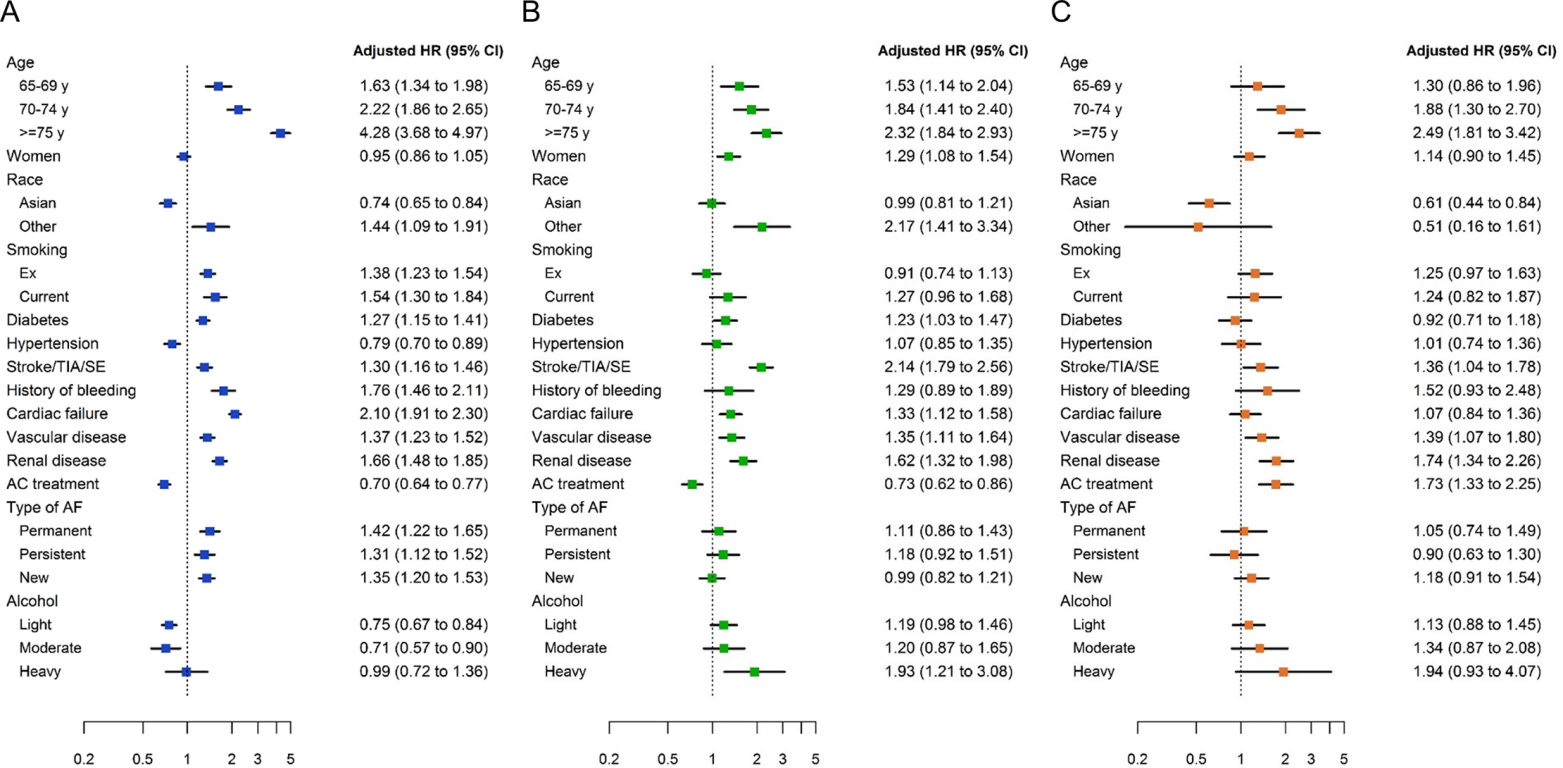
**Adjusted hazard ratios for 2-year outcomes according to baseline characteristics and anticoagulant treatment: (A) all-cause mortality; (B) stroke/systemic embolism; (C) major bleeding.** ‘Anticoagulant treatment’ includes both vitamin K antagonists and non-vitamin K antagonist oral anticoagulants. For race, ‘Asian’ includes Asian (not Chinese) and Chinese, and ‘other’ includes Afro-Caribbean, mixed/other, and unwilling to declare/not recorded. Reference groups, from top: <65 years, men, Caucasian/Hispanic/Latino, never smoker, no history of disease (for diabetes, hypertension, stroke/TIA/SE, history of bleeding, cardiac failure, vascular disease, and renal disease), no AC treatment, paroxysmal AF, alcohol abstinence. Hazard ratios were adjusted for all variables in the model. AC, anticoagulant; AF, atrial fibrillation; CI, confidence interval; HR, hazard ratio; SE, systemic embolism; TIA, transient ischaemic attack.

After adjustment, the following baseline variables were found to be significantly associated with a higher risk of death: age, ‘other race’ (vs Caucasian/Hispanic/Latino), smoking, diabetes mellitus, history of stroke/TIA/SE, history of bleeding, cardiac failure, VascD, moderate-to-severe renal disease, and non-paroxysmal forms of AF (Fig 1A). The variables associated with a higher risk of stroke/SE were: age, female sex, ‘other race’ (vs Caucasian/Hispanic/Latino), history of stroke/TIA/SE, CHF, VascD, moderate-to-severe renal disease, and heavy alcohol consumption (Fig 1B). Age, VascD, moderate-to-severe renal disease, and AC treatment were independently associated with a higher risk of major bleeding (Fig 1C). In addition, we observed a trend of increased risk of major bleeding with increasing alcohol consumption (p<0.0001), and a substantial but not statistically significant increase in bleeding risk with previous or current smoking.

Asians had significantly lower risks of death and major bleeding compared with Caucasian/Hispanic/Latino patients, but they also had a lower rate of comorbidities, particularly CKD (7.5% vs 11.09%, p<0.0001), and a significantly lower age (median [IQR] 69 [60–70] vs 72 [64–79]; p<0.0001). AC treatment was associated with lower risks of death and stroke/SE and a higher risk of major bleeding. History of hypertension and pattern of AF were not associated with a higher risk of stroke/SE.

The rates of death, stroke/SE, and major bleeding increased progressively with increasing grades of the CHA_2_DS_2_-VASc scoring scheme (Table 6).

**Table 6:** Crude event rates during 2-year follow-up according to CHA_2_DS_2_-VASc score.

Event	CHA_2_DS_2_-VASc 0–1 (n = 4694)	CHA_2_DS_2_-VASc 2 (n = 5539)	CHA_2_DS_2_-VASc 3 (n = 6753)	CHA_2_DS_2_-VASc 4+ (n = 11,642)
**Stroke/SE**				
n (%)	44 (0.9)	76 (1.4)	136 (2.0)	395 (3.4)
Rate per 100 person-years (95% CI)	0.52 (0.38; 0.69)	0.75 (0.60; 0.94)	1.11 (0.94; 1.32)	1.95 (1.77; 2.15)
**Major bleeding**				
n (%)	26 (0.6)	52 (0.9)	85 (1.3)	203 (1.7)
Rate per 100 person-years (95% CI)	0.30 (0.21; 0.45)	0.51 (0.39; 0.67)	0.69 (0.56; 0.86)	1.00 (0.87; 1.14)
**All-cause mortality**				
n (%)	123 (2.6)	208 (3.8)	391 (5.8)	1263 (10.8)
Rate per 100 person-years (95% CI)	1.44 (1.20; 1.71)	2.05 (1.79; 2.34)	3.17 (2.87; 3.50)	6.14 (5.81; 6.49)

CHA_2_DS_2_-VASc, cardiac failure, hypertension, age ≥75 (doubled), diabetes, stroke (doubled)-vascular disease, age 65–74, and sex category (female); CI, confidence interval; SE, systemic embolism.

## Discussion

Many reports have identified the variables associated with outcomes in AF, both in terms of stroke/SE and bleeding, but less frequently death and its causes [12–16]. This study is novel in addressing the variables that influence the risks of all three major outcome measures in a large, global, prospective registry of newly diagnosed AF. It confirms prior observations from a report based on a smaller cohort of the GARFIELD-AF registry with similar baseline characteristics and follow-up. We found that death was the most frequent adverse event, occurring at threefold the rate of stroke/SE and fivefold the rate of major bleeding [3]. Cardiovascular and non-cardiovascular causes of death occurred at a similar rate. Stroke accounted for less than 5% of all deaths. The most frequent causes of death were CHF, sudden death, ACS, malignancy, respiratory failure, and sepsis. Newly diagnosed AF appears to be both a marker of unfavourable outcome linked to underlying comorbidities and a worsening factor of some comorbidities such as CHF, ACS, and respiratory failure, which might be affected by the haemodynamic alterations and the embolic complications linked to AF [3, 17].

**Fig 2:**
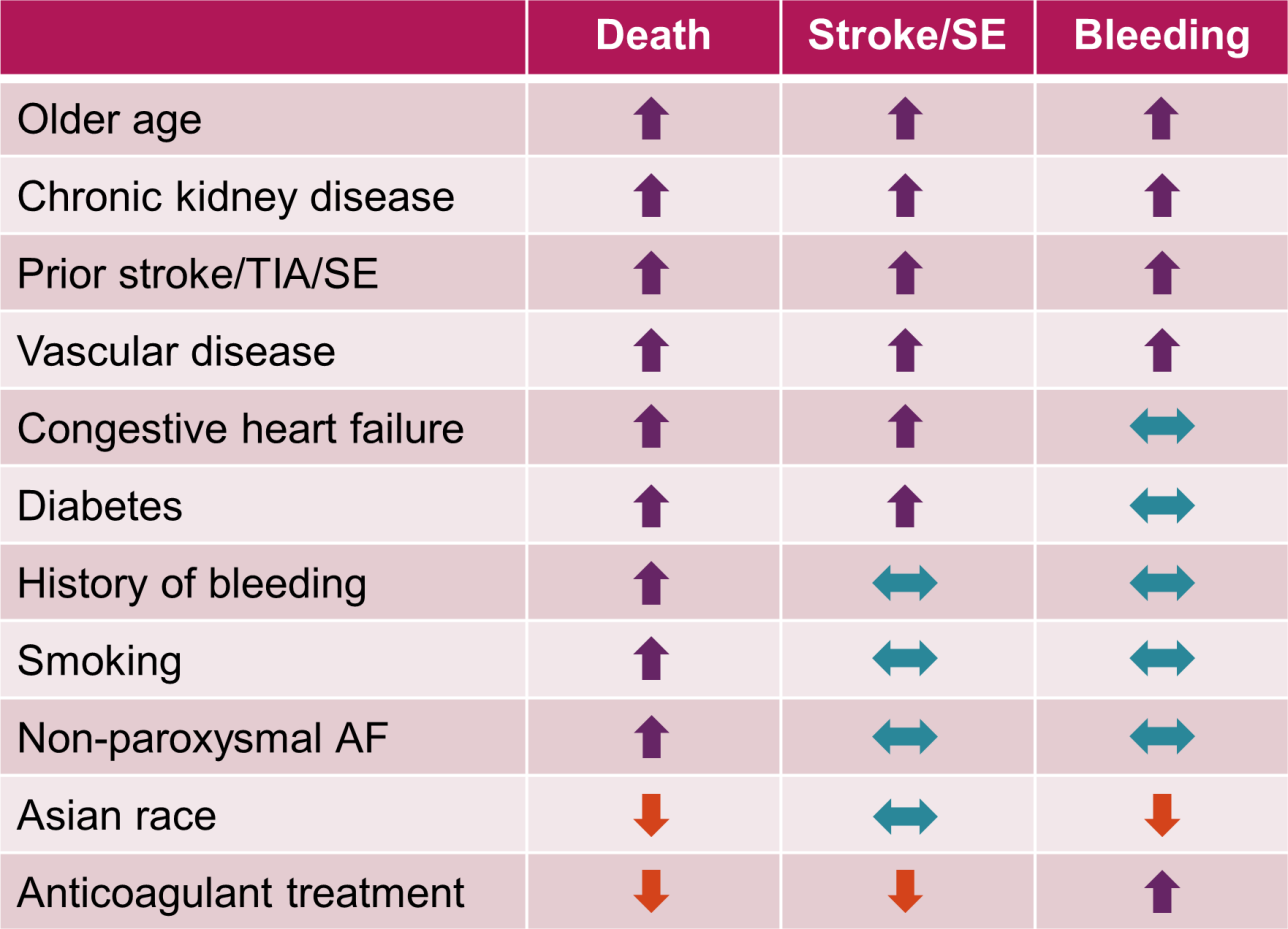
Relationships between variables and clinical outcomes during 2-year follow-up. Key to symbols used: upwards arrow indicates increased risk, downwards arrow indicates reduced risk, double-headed arrow indicates no change in risk. AF, atrial fibrillation; SE, systemic embolism; TIA, transient ischaemic attack.

The relationships between different variables and the risks of all-cause mortality, stroke/SE, and major bleeding are summarised in Fig 2. Four variables were associated with higher risks of all three major outcome measures, namely older age, history of stroke/TIA, VascD, and CKD. The first three variables are established predictors of stroke, and are components of the most widely used risk assessment scheme CHA_2_DS_2_-VASc. Though CKD is not a component of the CHA_2_DS_2_-VASc score, it has a strong influence on the risks of stroke/SE, bleeding, and death [12, 14, 16, 18–20]. CHF, also a component of CHA_2_DS_2_-VASc, was associated with higher risks of both death and stroke/SE. Two variables were associated with a higher risk of death only, namely smoking and history of bleeding.

Non-paroxysmal forms of AF were also associated with a higher risk of death, but not of stroke/SE compared with paroxysmal AF. In previous reports from randomised controlled trials and registries, non-paroxysmal forms of AF were consistently reported to be associated with a higher risk of death, but with various findings as regards the risk of stroke/SE [13, 21–24]. Diabetes was also associated with a higher risk of death but was only marginally associated with a higher risk of stroke/SE. Adequate blood glucose balance, shown to prevent the occurrence of microvascular complications and to a lesser extent cardiovascular complications, may explain the lack of association of diabetes with the risk of stroke/SE [25, 26].

Some variables considered to be risk factors were only marginally associated with the risk of events or had a neutral effect, for example, history of hypertension. One may assume that history of hypertension as reported by the investigators in over 70% of the population implies that adequate control of blood pressure was achieved in these patients. We may hypothesise that this may have prevented remodelling of the heart chambers. Interestingly, 7.5% of patients included in GARFIELD-AF had uncontrolled hypertension (systolic blood pressure >160 mmHg). This subset of patients had a higher risk of stroke/SE but not of death or bleeding compared to patients without high blood pressure (HR 1.73, 95% CI 1.37 to 2.18).

AC was associated with substantially lower risks of all-cause death (30% lower risk in AC patients compared with non-AC patients) and stroke/SE (28% lower risk in patients receiving AC compared with those not receiving AC), and with a 52% higher risk of bleeding compared with no AC treatment. Reduction in mortality in association with anticoagulation may have been due, in part, to prevention of thromboembolism in patients with CHF, sepsis, respiratory failure, or cancer [3].

In contrast, Asian race was associated with lower risks of death and bleeding, without an excess of stroke/SE compared with non-Asian races. Conflicting observations were previously reported on this issue with the risks of death, stroke/SE, and bleeding found to be identical or higher in Asians compared to non-Asians [27, 28]. In this report, these observations may be due to confounding factors despite adjustment, as these patients were at lower risk than non-Asian patients, as shown by their younger median age, lower rate of comorbidities, particularly CKD, and lower CHA_2_DS_2_-VASc score. In addition, in some Asian countries, physicians routinely target a lower INR than in other regions, resulting in a significantly lower risk of bleeding compared with non-Asian patients, yet with no excess risk of stroke/SE [29, 30].

Overall, two messages arise from this report. Firstly, several variables are linked to the risk of one or more outcome measures. So far, the therapeutic approach recommended for the management of AF emphasises rhythm and/or rate control and anticoagulation administered in at-risk patients without contraindication. The decision for treatment is based on clinical judgment and assessment of the risks of stroke/SE and bleeding using clinical risk scores, most commonly CHA_2_DS_2_-VASc and HAS-BLED. Biomarker-based scores like the recently described ABC score [31] cannot be used in a non-interventional registry, as blood sampling is an intervention. In addition, this approach does not consider the most important risk for patients with AF, namely the risk of death. Therefore, the tools used to assess risks need to be revisited as many variables shown to have an impact on outcome are not included in the commonly used risk scores, and because the risk of death is not considered. For that reason, a new scoring system addressing the risks of all three major outcome measures at once, namely death, stroke/SE and bleeding, derived from a larger cohort may prove useful for clinicians. Such a tool is under development using a larger GARFIELD-AF population as derivation cohort and will be tested on an external validation cohort.

Secondly, this study shows that the comorbidities that strongly affect outcomes are undertreated in at least one-third of patients with newly diagnosed AF. All comorbidities shown to have an impact on outcomes should be addressed. In this regard, aggressive management of CHF, CKD, and VascD and their risk factors, as well as smoking and drinking cessation, should be strongly advocated. Strikingly, patients with comorbidities associated with the risk of two or all three outcome measures, namely CHF, VascD, and CKD, are undertreated if we consider the rates of prescription of guideline-recommended therapies. In patients with CHF, depending on the New York Heart Association (NYHA) class, up to four drugs may be needed [5]. Our data show that irrespective of the NYHA class, at least half of the patients were receiving a maximum of two drugs. In patients with VascD who should receive long term four pharmacological classes, more than half of them were receiving a maximum of two [32]. Undertreatment has commonly been reported in various settings [33–37]. The situation for CKD is more difficult to analyse, as the prescription of ACE/ARB is not unanimously recommended and implemented, and is mostly driven by comorbidities [38, 39]. On the other hand, implementation of guideline-recommended therapies was shown to improve outcomes, particularly in CHF and ACS [40–44]. Whether aggressive management of comorbidities in new-onset AF has a favourable impact on outcomes is not yet known. Only controlled randomised trials comparing strict and lenient management strategies, and to some extent real-life prospective registries such as GARFIELD-AF may help to answer this question.

## Study limitations

We focused on three variables only when assessing the prescription rates of guideline-recommended therapies. We cannot assume that our observations are valid for other variables. We did not differentiate between CHF with preserved or reduced ejection fraction. In addition, the collection of data on drug prescriptions in patients with CHF, VascD, and CKD may be suboptimal since GARFIELD-AF is a study on patients with AF, and not specifically on these entities.

## Conclusions

Death is the most frequent adverse event in newly diagnosed AF, occurring at threefold the rate of stroke/SE and fivefold the rate of major bleeding. Many modifiable variables have an influence on the risk of one or more major outcome measures, particularly CHF, VascD, and CKD, whose management seems suboptimal as regards the prescription of guideline-recommended drugs in routine practice. Based upon these observations, we advocate for comprehensive management of newly diagnosed AF, particularly the implementation of guideline-recommended therapies in CHF, VascD, and CKD, in addition to anticoagulation. Registries like GARFIELD-AF may help to elucidate the causes of under treatment and to assess the impact on outcomes of aggressive management of the most influential comorbidities.

## Supporting information

S1 Appendix GARFIELD-AF registry investigators. (DOCX)

S2 Appendix Central ethics committees and regulatory authorities. (XLSX)
