# Supplementary material for: Risk factors for death, stroke, and bleeding in 28,628 patients from the GARFIELD-AF registry: Rationale for comprehensive management of atrial fibrillation
Source: PLoS One. 2018 Jan 25;13(1):e0191592. doi: 10.1371/journal.pone.0191592 (PMC5784935; doi:10.1371/journal.pone.0191592)
Supplement: S1 Appendix — (DOCX) [file pone.0191592.s001.docx]

**Global Steering Committee**

Ajay K. Kakkar (UK) (Chair), Jean-Pierre Bassand (France), A. John Camm (UK), David A. Fitzmaurice (UK), Samuel Z. Goldhaber (USA), Shinya Goto (Japan), Sylvia Haas (Germany), Werner Hacke (Germany), Lorenzo G. Mantovani (Italy), Frank Misselwitz (Germany), Karen S. Pieper (USA), Alexander G.G. Turpie (Canada), Martin van Eickels (Germany), Freek W.A. Verheugt (the Netherlands).

**Publications Committee**

A. John Camm (UK) (Chair), Jean-Pierre Bassand (France), Samuel Z. Goldhaber (USA), Sylvia Haas (Germany), Gloria Kayani (UK), Lorenzo G. Mantovani (Italy).

**Audit Committee**

Keith A.A. Fox (UK), Bernard J. Gersh (USA).

**GARFIELD-AF National Coordinators**

Hector Lucas Luciardi (Argentina), Harry Gibbs (Australia), Marianne Brodmann (Austria), Frank Cools (Belgium), Antonio Carlos Pereira Barretto (Brazil), Stuart J. Connolly, Alex Spyropoulos, John Eikelboom (Canada), Ramon Corbalan (Chile), Dayi Hu (China), Petr Jansky (Czech Republic), Jørn Dalsgaard Nielsen (Denmark), Hany Ragy (Egypt), Pekka Raatikainen (Finland), Jean-Yves Le Heuzey (France), Harald Darius (Germany), Matyas Keltai (Hungary), Sanjay Kakkar and Jitendra Pal Singh Sawhney (India), Giancarlo Agnelli and Giuseppe Ambrosio (Italy), Yukihiro Koretsune (Japan), Carlos Jerjes Sánchez Díaz (Mexico), Hugo Ten Cate (the Netherlands), Dan Atar (Norway), Janina Stepinska (Poland), Elizaveta Panchenko (Russia), Toon Wei Lim (Singapore), Barry Jacobson (South Africa), Seil Oh (South Korea), Xavier Viñolas (Spain), Marten Rosenqvist (Sweden), Jan Steffel (Switzerland), Pantep Angchaisuksiri (Thailand), Ali Oto (Turkey), Alex Parkhomenko (Ukraine), Wael Al Mahmeed (United Arab Emirates), David Fitzmaurice (UK), Samuel Z. Goldhaber (USA).

**GARFIELD-AF National Investigators**

***Asia***

*China:* D.Y. Hu, K.N. Chen, Y.S. Zhao, H.Q. Zhang, J.Z. Chen, S.P. Cao, D.W. Wang, Y.J. Yang, W.H. Li, Y.H. Yin, G.Z. Tao, P. Yang, Y.M. Chen, S.H. He, Y. (Ying) Wang, Y. (Yong) Wang, G.S. Fu, X. Li, T.G. Wu, X.S. Cheng, X.W. Yan, R.P. Zhao, M.S. Chen, L.G. Xiong, P. Chen, Y. Jiao, Y. Guo, L. Xue, F.Z. Wang, H. Li, Z.M. Yang, C.L. Bai, J. Chen, J.Y. Chen, X. Chen, S. Feng, Q.H. Fu, X.J. Gao, W.N. Guo, R.H. He, X.A. He, X.S. Hu, X.F. Huang, B. Li, J. Li, L. Li, Y.H. Li, T.T. Liu, W.L. Liu, Y.Y. Liu, Z.C. Lu, X.L. Luo, T.Y. Ma, J.Q. Peng, X. Sheng, X.J. Shi, Y.H. Sun, G. Tian, K. Wang, L. Wang, R.N. Wu, Q. Xie, R.Y. Xu, J.S. Yang, L.L. Yang, Q. Yang, Y.J. Yang, Y. Ye, H.Y. Yu, J.H. Yu, T. Yu, H. Zhai, Q. Zhan, G.S. Zhang, Q. Zhang, R. Zhang, Y. Zhang, W.Y. Zheng, B. Zhou, Z.H. Zhou, X.Y. Zhu.

*India:* S. Kakkar, J.P.S. Sawhney, P. Jadhav, R. Durgaprasad, A.G. Ravi Shankar, R.K. Rajput, K. Bhargava, R. Sarma, A. Srinivas, D. Roy, U.M. Nagamalesh, M. Chopda, R. Kishore, G. Kulkarni, P. Chandwani, R.A. Pothiwala, M. Padinhare Purayil, S. Shah, K. Chawla, V.A. Kothiwale, B. Raghuraman, G. Vijayaraghavan, V.M. Vijan, G. Bantwal, V. Bisne, A. Khan, J.B. Gupta, S. Kumar, D. Jain, S. Abraham, D. Adak, A. Barai, H. Begum, P. Bhattacharjee, M. Dargude, D. Davies, B. Deshpande, P. Dhakrao, V. Dhyani, S. Duhan, M. Earath, A. Ganatra, S. Giradkar, V. Jain, R. Karthikeyan, L. Kasala, S. Kaur, S. Krishnappa, A. Lawande, B. Lokesh, N. Madarkar, R. Meena, P. More, D. Naik, K. Prashanth, M. Rao, N.M. Rao, N. Sadhu, D. Shah, M. Sharma, P. Shiva, S. Singhal, S. Suresh, V. Vanajakshamma, S.G. Panse.

*Japan:* Y. Koretsune, S. Kanamori, K. Yamamoto, K. Kumagai, Y. Katsuda, K. Sadamatsu, F. Toyota, Y. Mizuno, I. Misumi, H. Noguchi, S. Ando, T. Suetsugu, M. Minamoto, H. (Hiroshi) Oda, K. Shiraishi, S. Adachi, K. Chiba, H. Norita, M. Tsuruta, T. Koyanagi, K. Yamamoto, H. Ando, T. Higashi, K. Okada, S. Azakami, S. Komaki, K. Kumeda, T. Murayama, J. Matsumura, Y. Oba, R. Sonoda, K. Goto, K. Minoda, Y. Haraguchi, H. Suefuji, H. Miyagi, H. Kato, T. (Tadashi) Nakamura, T. (Tsugihiro) Nakamura, H. Nandate, R. Zaitsu, Y. (Yoshihisa) Fujiura, A. Yoshimura, H. Numata, J. Ogawa, H. Tatematsu, Y. Kamogawa, K. Murakami, Y. Wakasa, M. Yamasawa, H. Maekawa, S. Abe, H. Kihara, S. Tsunoda, K. (Katsumi) Saito, K. (Kazuyuki) Saito, T. Fudo, K. Obunai, H. Tachibana, I. Oba, T. Kuwahata, S. Higa, M. Gushiken, T. Eto, H. Yoshida, D. Ikeda, Y. (Yoshitake) Fujiura, M. Ishizawa, M. Nakatsuka, K. Murata, C. Ogurusu, M. Shimoyama, M. Akutsu, I. Takamura, F. Hoshino, N. Yokota, T. Iwao, K. Tsuchida, M. Takeuchi, Y. Hatori, Y. Kitami, Y. (Yoichi) Nakamura, R. Oyama, M. Ageta, H. (Hiroyuki) Oda, Y. Go, K. Mishima, T. Unoki, S. Morii, Y. (Yuhei) Shiga, H. Sumi, T. Nagatomo, K. Sanno, K. Fujisawa, Y. Atsuchi, T. Nagoshi, T. Seto, T. Tabuchi, M. Kameko, K. Nii, K. Oshiro, H. Takezawa, S. Nagano, N. Miyamoto, M. Iwaki, Y. (Yuichiro) Nakamura, M. Fujii, M. Okawa, M. (Masahiko) Abe, M. (Masatake) Abe, M. (Mitsunori) Abe, T. Saito, T. Mito, K. Nagao, J. Minami, T. Mita, I. Sakuma, T. Taguchi, S. Marusaki, H. Doi, M. Tanaka, T. Fujito, M. Matsuta, T. Kusumoto, S. Kakinoki, K. Ashida, N. Yoshizawa, J. Agata, O. Arasaki, M. Manita, M. Ikemura, S. Fukuoka, H. Murakami, S. Matsukawa, Y. Hata, T. Taniguchi, T. Ko, H. Kubo, M. Imamaki, M. Akiyama, M. Inagaki, H. Odakura, T. Ueda, Y. Katsube, A. Nakata, H. Watanabe, M. Techigawara, M. Igarashi, K. Taga, T. Kimura, S. Tomimoto, M. Shibuya, M. Nakano, K. Ito, T. Seo, S. Hiramitsu, H. Hosokawa, M. Hoshiai, M. Hibino, K. Miyagawa, H. (Hajime) Horie, N. Sugishita, Y. (Yukio) Shiga, A. Soma, K. Neya, T. (Tetsuro) Yoshida, T. (Tomoki) Yoshida, M. Mizuguchi, M. Ishiguro, T. Minagawa, M. Wada, H. Mukawa, F. Okuda, S. Nagasaka, Y. Abe, S. (Sen) Adachi, S. (Susumu) Adachi, T. Adachi, K. Akahane, T. Amano, K. Aoki, T. Aoyama, H. Arai, S. Arima, T. Arino, H. Asano, T. Asano, J. Azuma, T. Baba, T. Betsuyaku, H. Chibana, H. Date, J. Doiuchi, Y. Emura, M. Endo, Y. Fujii, R. Fujiki, A. Fujisawa, Y. Fujisawa, T. Fukuda, T. Fukui, N. Furukawa, T. Furukawa, W. Furumoto, T. Goto, M. Hamaoka, N. Hanazono, K. Hasegawa, T. Hatsuno, Y. Hayashi, K. Higuchi, K. Hirasawa, H. Hirayama, M. Hirose, S. Hirota, M. Honda, H. (Hideki) Horie, T. Ido, O. Iiji, H. Ikeda, K. Ikeda, K. Ikeoka, M. Imaizumi, H. Inaba, T. Inoue, F. Iseki, A. Ishihara, N. Ishioka, N. Ito, T. Iwase, H. Kakuda, J. Kamata, H. Kanai, H. Kanda, M. Kaneko, H. Kano, T. Kasai, T. Kato, Y. Kato, Y. Kawada, K. Kawai, K. Kawakami, S. Kawakami, T. Kawamoto, S. Kawano, J. Kim, T. Kira, H. Kitazawa, H. Kitazumi, T. Kito, T. Kobayashi, T. Koeda, J. Kojima, H. Komatsu, I. Komatsu, Y. Koshibu, T. Kotani, T. Kozuka, Y. Kumai, T. Kumazaki, I. Maeda, K. Maeda, Y. Maruyama, S. Matsui, K. Matsushita, Y. Matsuura, K. Mineoi, H. Mitsuhashi, N. Miura, S. Miyaguchi, S. Miyajima, H. Miyamoto, A. Miyashita, S. Miyata, I. Mizuguchi, A. Mizuno, T. Mori, O. Moriai, K. Morishita, O. Murai, S. (Sho) Nagai, S. (Shunichi) Nagai, E. Nagata, H. Nagata, A. Nakagomi, S. Nakahara, M. Nakamura, R. Nakamura, N. Nakanishi, T. Nakayama, R. Nakazato, T. Nanke, J. Nariyama, Y. Niijima, H. Niinuma, Y. Nishida, Y. Nishihata, K. Nishino, H. Nishioka, K. Nishizawa, I. Niwa, K. Nomura, S. Nomura, M. Nozoe, T. Ogawa, N. Ohara, M. Okada, K. Okamoto, H. Okita, M. Okuyama, H. Ono, T. Ono, Y. Onuki Pearce, S. Oriso, A. Ota, E. Otaki, Y. Saito, H. Sakai, N. Sakamoto, Y. Sakamoto, Y. Samejima, Y. Sasagawa, H. Sasaguri, A. Sasaki, T. Sasaki, K. (Kazuki) Sato, K. (Kiyoharu) Sato, M. Sawano, S. Seki, Y. Sekine, Y. Seta, K. Sezaki, N. Shibata, Y. Shiina, H. Shimono, Y. Shimoyama, T. Shindo, H. Shinohara, R. Shinohe, T. Shinozuka, T. Shirai, T. Shiraiwa, Y. Shozawa, T. Suga, C. Sugimoto, K. (Kazuo) Suzuki, K. (Keita) Suzuki, S. (Shu) Suzuki, S. (Shunji) Suzuki, S. (Susumu) Suzuki, Y. Suzuki, M. Tada, A. Taguchi, T. Takagi, Y. Takagi, K. Takahashi, S. Takahashi, H. Takai, C. Takanaka, S. Take, H. Takeda, K. Takei, K. Takenaka, T. Tana, G. Tanabe, K. Taya, H. Teragawa, S. Tohyo, S. Toru, Y. Tsuchiya, T. Tsuji, K. Tsuzaki, H. Uchiyama, O. Ueda, T. Ueda, Y. Ueyama, N. Wakaki, T. Wakiyama, T. Washizuka, M. Watanabe, T. Yamada, T. Yamagishi, H. Yamaguchi, K. (Kenichi) Yamamoto, K. (Kentaro) Yamamoto, K. (Kunihiko) Yamamoto, T. Yamamoto, M. Yamaura, M. Yamazoe, K. Yasui, Y. Yokoyama, K. Yoshida.

*Singapore:* T.W. Lim, C.K. Ching, C.G. Foo, J.H. Chow, D.D. Chen, F.R. Jaufeerally, Y.M. Lee, H. Li, G. Lim, W.T. Lim, S. Thng, S.Y. Yap, C. Yeo.

*South Korea:* S. Oh, H.N. Pak, J.-B. Kim, J.H. Kim, S.-W. Jang, D.H. Kim, J. Kim, D.R. Ryu, S.W. Park, D.-K. Kim, D.J. Choi, Y.S. Oh, M.-C. Cho, S.-H. Kim, H.-K. Jeon, D.-G. Shin, J.S. Park, H.K. Park, S.-J. Han, J.H. Sung, J.-G. Cho, G.-B. Nam, Y.K. On, H.E. Lim, J.J. Kwak, T.-J. Cha, T.J. Hong, S.H. Park, J.H. Yoon, N.-H. Kim, K.-S. Kim, B.C. Jung, G.-S. Hwang, C.-J. Kim, J.S. Park, D.B. Kim, J.J. Ahn, H.J. An, H. Bae, A.L. Baek, W.J. Chi, E.A. Choi, E.H. Choi, H.K. Choi, H.S. Choi, S. Han, E.S. Heo, K.O. Her, S.W. Hwang, E.M. Jang, H.-S. Jang, S. Jang, H.-G. Jeon, S.R. Jeon, Y.R. Jeon, H.K. Jeong, I.-A. Jung, H.J. (Hyeon Jeong) Kim, H.J. (Hyun Ju) Kim, J.S. (Ji Seon) Kim, J.S. (Jung Sook) Kim, J.A. Kim, K.T. Kim, M.S. Kim, S.H. (Sang Hee) Kim, S.H. (Sang Hyun) Kim, Y.-I. Kim, C.S. Lee, E.H. Lee, G.H. Lee, H.Y. Lee, H.-Y. Lee, K.H. Lee, K.R. Lee, M.S. Lee, M.-Y. Lee, R.W. Lee, S.E. Lee, S.H. Lee, S. Lee, W.Y. Lee, I.K. Noh, A.R. Park, B.R. Park, H.N. Park, J.H. Park, M. Park, Y. Park, S.-Y. Seo, J. Shim, J.H. Sim, Y.M. Sohn, W.S. Son, Y.S. Son, H.J. Song, H.K. Wi, J.J. Woo, S. Ye, K.H. Yim, K.M. Yoo, E.J. Yoon, S.Y. Yun.

*Thailand:* P. Angchaisuksiri, S. Chawanadelert, P. Mongkolwongroj, K. Kanokphatcharakun, S. Cheewatanakornkul, T. Laksomya, S. Pattanaprichakul, T. Chantrarat, S. Rungaramsin, S. Silaruks, W. Wongcharoen, K. Siriwattana, K. Likittanasombat, P. Katekangplu, W. Boonyapisit, D. Cholsaringkarl, B. Chatlaong, P. Chattranukulchai, Y. Santanakorn, P. Hutayanon, P. Khunrong, T. Bunyapipat, S. Jai-Aue, P. Kaewsuwanna, P. Bamungpong, S. Gunaparn, S. Hongsuppinyo, R. Inphontan, R. Khattaroek, K. Khunkong, U. Kitmapawanont, C. Kongsin, B. Naratreekoon, S. Ninwaranon, J. Phangyota, A. Phrommintikul, P. Phunpinyosak, K. Pongmorakot, S. Poomiphol, N. Pornnimitthum, S. Pumprueg, S. Ratchasikaew, K. Sanit, K. Sawanyawisuth, B. Silaruks, R. Sirichai, A. Sriwichian, W. Suebjaksing, P. Sukklad, T. Suttana, A. Tangsirira, O. Thangpet, W. Tiyanon, Y. Vorasettakarnkij, T. Wisaratapong, W. Wongtheptien, A. Wutthimanop, S. Yawila.

*Turkey*: A. Oto, A. Altun, I. Ozdogru, K. Ozdemir, O. Yilmaz, A. Aydinlar, M.B. Yilmaz, E. Yeter, Z. Ongen, M. Cayli, H. Pekdemir, M. Ozdemir, M. Sucu, T. Sayin, M. Demir, H. Yorgun, M. Ersanli, E. Okuyan, D. Aras, H. Abdelrahman, O. Aktas, D. Alpay, F. Aras, M.F. Bireciklioglu, S. Budeyri, M. Buyukpapuc, S. Caliskan, M. Esen, M.A. Felekoglu, D. Genc, B. Ikitimur, E.B. Karaayvaz, S. Kılıç Karataş, S. Okutucu, E. Ozcelik, A. Quisi, H. Sag, L. Sahiner, B.Y. Sayin, T. Seker, D. Uzun Alkan, E. Yildirim, R. Yildirim, F. Yilmaz, V. Yuksekdag.

***Central/South America***

*Argentina:* H.L. Luciardi, N. Vensentini, A.C. Ingaramo, G.A. Sambadaro, V. Fernandez Caputi, S.G. Berman, P. Dragotto, A.J. Kleiban, N. Centurion, G. Giacomi, R.A. Ahuad Guerrero, D. Conde, G. Zapata, L.A. Di Paola, J.L. Ramos, R.D. Dran, J. Egido, A.A. Fernandez, M.J. Fosco, S. Sassone, V.A. Sinisi, L.R. Cartasegna, M.A. Berli, O.A. Gomez Vilamajo, F. Ferroni, E.D. Alaguibe, A. Alvarez D'Amelio, C. Arabetti, L. Arias, J.A. Belardi, L. Bergesio, F. Berli, M. Berli, S. Borchowiec, C. Buzzetti, R. Cabrini, V. Campisi, A.L. Cappi, R. Carrizo, F. Colombo Berra, J.P. Costabel, O.J.A. Costamagna, A.A. Damonte, I.N. De Urquiza, F. Diez, M.F. Edén, M. Fanuele, F. Fernandez Voena, M. Foa Torres, C. Funosas, M.P. Giacomi, C.H. Gimenez, E.P. Gurfinkel, M. de L.M. Had, V. Hansen, A.D. Hrabar, M. Ingratta, A. Lopez, G. Maehara, L. Maffei, A. Martinelli, C. Martinelli, J. Matkovich, B. Mautner, A. Meirino, R. Munguia, A. Navarro, V. Novas, G. Perez Prados, J. Pontoriero, R.N. Potito, C. Ricotti, M.A. Rodriguez, F. Rolandi, M.E. Said Palladino, M. Salinger, L.S. Sanziani, P.O. Schygiel, A. Sossich, J.F. Tinto, L. Tonelli, A.L. Tufare, M. Vallejo, M.E. Yunis, M. Zillo, F.J. Zurbrigk.

*Brazil:* A.C.P. Barretto, D.C. Sobral Filho, J. Jaber, D. Armaganijan, J. Faria Neto, A. Steffens, W. Kunz Sebba Barroso de Souza, J.D. de Souza Neto, J.M. Ribeiro, M. Silveira Teixeira, P.R. Ferreira Rossi, L. Pires, D. Moreira, J.C. Moura Jorge, A. Menezes Lorga Filho, L.C. Bodanese, M. Westerlund Montera, C.H. Del Carlo, T. Da Rocha Rodrigues, F.A. Alves da Costa, A. Lopes, R. Lopes, G.R. Araújo, E.R. Fernandes Manenti, J.F. Kerr Saraiva, J.C. Ferreira Braga, A. Negri, L. Souto, C. Moncada, D. Bertolim Precoma, F. Roquette, G. Reis, R.A. Ramos Filho, E. Lanna Figueiredo, R. Vieira Botelho, C. Munhoz da Fontoura Tavares, C.R. Costantini Frack, J. Abdalla Saad, H.C. Finimundi, C. Pisani, D. Chemello, M. Pereira Martins, C.C. Broilo França, F. Alban, G.B. Aranha Rosito, J.B. de Moura Xavier Moraes Junior, R.T. Tumelero, L. Nigro Maia, R. Simões de Almeida, N.C. do Carmo Borges, L.G. Gomes Ferreira, P. Agliardi, J. Alves de Oliveira Gomes, V. Araujo, M. Arruda Nakazone, T. Barbosa, S. Barroso, E. Belisario Falchetto, H. Bellotti Lopes, M.A. Benez Teixeira Lemos, G. Biazus, L. Borges Queiroz, F.E. Camazzola, M. Caporale, S. Cardoso Boscato, F. Chieza, M.O. Chokr, R. Clemente Mingireanov, N. Codonho Góes, C. Correa, M. Costa, C. Costantini Ortiz, L.S. da Silva, F. da Silva Paulitsch, J.A. da Silveira, E. Daros, G.R. de Araújo, M.I. Del Monaco, C. Dias, M.A. Dias, A.P. Drummond Wainstein, P. Ely Pizzato, D.C. Esteves, P. Fabri, T. Félix Lorenzato Fonseca, E. Fernandes, C. Fonseca, C.R. Frack Costantini, R. Franchin Ferraz, F. Freire, P. Gottardo, D. Guanaes, S. Guizzardi, E. Hettwer Magedanz, F. Igansi, F. Jannuzzi, G. Junior, D. Komar, E.G. Lino, D. Lopes, O. Lourenço da Silva Júnior, E. Lustosa, A.P. Macagnan, M.C. Marinho, M. Mazzoni, G. Melo, L. Mortari, O.M.C.C. Mouco, C. Nanzer Vital, C. Ormundo, S. Oss Emmer, E. Palmegiani, R. Pavani, L. Pereira, V.L. Pereira, R. Perreira, S. Poletti, S.C. Quaia Fortunato, C. Queirantes, N. Ramos Pereira, R.L. Rech, S. Ribeiro, A. Rodrigues, H. Roesch, T. Ruaro Reichert, D. Santos, I. Santos, M. Santos, M.V. Seroqui, S. Silva, L. Soares, L. Spolaor, C. Stoll, N. Toazza Duda, L. Trama, B. Unterkircher, M.V. Valois, T. Vargas, T. Viana, C. Vicente, L. Vidal Armaganijan, R. Vieira Homem, L.G. Vieira Torres, L. Vila Boas, F. Villaça Guimarães Filho.

*Chile:* R. Corbalan, G. Eggers, C. Bugueño Gutiérrez, G. Arriagada, S. Potthoff Cardenas, B.A.J. Stockins Fernandez, C. Conejeros, C. Houzvic, P. Marin Cuevas, H. Montecinos, A. Forero, F. Lanas, M. Larico Gómez, G. Charme Vilches, C. Rey, C. Astudillo, J. Aguilar, Y. Campisto, C. Lara, E. Molina, J. Munoz Oyarzon, V. Olguin, M. Vergara, C. Villan.

*Mexico:* C.J. Sánchez Díaz, J. Illescas Diaz, R. Leal Cantú, M.G. Ramos Zavala, R. Cabrera Jardines, N. Espinola Zavaleta, S. Villarreal Umaña, E. López Rosas, G. Llamas Esperón, G. Pozas, E. Cardona Muñoz, N. Matadamas Hernández, A. Leyva Rendón, N. García Hernández, M. de los Ríos Ibarra, L. Virgen Carrillo, D. López Villezca, C. Hernández Herrera, J.J. López Prieto, R. Gaona Rodríguez, E. Villeda Espinosa, D. Flores Martínez, J. Velasco Barcena, R. Yong, I. Rodríguez Briones, J.L. Leiva Pons, H. Álvarez López, R. Olvera Ruiz, C. Díaz de la Vega, C. Cantú Brito, E. Chuquiure Valenzuela, R. Reyes-Sanchez, A. Bazzoni Ruiz, O. Nandayapa Flores, M. Benavides Gonzalez, R. Arriaga Nava, J.D. Morales Cerda, O. Fierro Fierro, P. Fajardo Campos, T.A.A. Alfaro, S. Altamirano Bellorin, R. Avena, M. Chavarria, I. Espinosa, F. Flores Silva, R.H. Garcia Nava, K. Godoy, E.J. Gonzalez Felix, C.L. Gonzalez Garcia, L.G. Gonzalez Salas, P. Guajardo, S. Hernandez Gonzalez, T. Izquierdo, M.C. Mancilla Ortiz, D. Martinez Vasquez, N. Mendoza, J. Morales, N. Nikitina, S. Ochoa Aybar, A. Ortiz, P. Padilla Macias, F. Perez, J.A. Perez Sanchez, S. Piña Toledano, M. Ramos Gonzalez, C. Rivera Ramos, V. Roa Castro, G. Romero Cardona, M. Ruiz Cornejo, A. Salinas, G. Santana, P. Sida Perez, A.C. Tovar Castaneda, R. Trujillo Cortes.

***Europe***

*Austria:* M. Brodmann, K. Lenz, H. Drexel, J. Foechterle, C. Hagn, A. Podczeck-Schweighofer, K. Huber, M. Winkler, B. Schneeweiß, A. Gegenhuber, W. Lang, S. Eichinger-Hasenauer, P. Kaserer, J. Sykora, H. Rasch, M. Pichler, E. Schaflinger, B. Strohmer, R. Breier, K.-M. Ebner, L. Eischer, F. Freihoff, A. Lischka-Lindner, T. Mark, A. Mirtl, A. Said, C. Stöcklöcker, B. Vogel, A. Vonbank, C. Wöhrer, D. Zanolin.

*Belgium:* F. Cools, G. Paparella, P. Vandergoten, J.-L. Parqué, L. Capiau, G. Vervoort, B. Wollaert, P. Desfontaines, G. Mairesse, T. Boussy, P. Godart, A. De Wolf, J. Voet, A. Heyse, G. Hollanders, W. Anné, J. Vercammen, P. Purnode, I. Blankoff, D. Faes, Y. Balthazar, M. Beutels, P. Maréchal, S. Verstraete, O. Xhaet, H. Striekwold, J. Thoeng, K. Hermans, B. Alzand, A.-K. Ascoop, F. Banaeian, A.-M. Barbuto, A.C. Billiaux, M. Blockmans, C. Bouvy, C. Brike, H. Capiau, T. Casier, A. Conde Y Bolado, D. De Cleen, M. De Coninck, M. de Vos, N. de Weerdt, M. Delforge, M. Delvigne, D. Denie, K. Derycker, E. Deweerd, F. Dormal, S. Drieghe, M. Everaert, T. Eykerman, E. Feys, M. Ghekiere, F. Gits, S. Hellemans, L. Helvasto, C. Jacobs, S. Lips, I. Mestdagh, J. Nimmegeers, V. Piamonte, P. Pollet, A. Postolache, M. Raepers, E. Raymenants, J. Richa, H. Rombouts, J. Salembier, C. Scheurwegs, O. Semeraro, N. Simons, C. Smessaert, W. Smolders, I. Stockman, S. Tahon, V. Thyssen, G. Tincani, F. Van Durme, D. Van Lier, H. Vandekerckhove, Y. Vandekerckhove, D. Vandenbroeck, A. Vandorpe, E. Vanhalst, B. Vanhauwaert, C. Vantomme, L. Vergauwen, H. Verloove, T. Vydt, T. Weyn.

*Czech Republic:* P. Jansky, P. Reichert, R. Spacek, V. Machova, E. Zidkova, O. Ludka, J. Olsr, L. Kotik, K. Plocova, B. Racz, R. Ferkl, J. Hubac, I. Kotik, Z. Monhart, H. Burianova, O. Jerabek, J. Pisova, I. Petrova, V. Dedek, M. Honkova, P. Podrazil, J. Spinar, J. Vitovec, M. Novak, J. Lastuvka, V. Durdil, P. Antonova, L. Bockova, J. Bultas, J. Chlumsky, L. Dastychova, T. Drasnar, J. Honek, M. Horejsi, V. Hubacova, L. Janska, I. Kopeckova, R. Kratochvilova, E. Krcova, R. Labrova, A. Lindourkova, J. Lipoldova, H. Lubanda, A. Ludkova, L. Mahdalikova, M. Majerníkova, D. Michalik, P. Potuznik, E. Prochazkova, A. Sulc, J. Sveceny, M. Valtova, M. Zidek, J. Zika.

*Denmark*: J. Nielsen, H. Nielsen, S. Husted, U. Hintze, S. Rasmussen, A. Bremmelgaard, J. Markenvard, J. Boerger, J. Solgaard, P. Simonsen, T. Loekkegaard, M. Bruun, J. Mertz, H. Domínguez, K. Skagen, K. Egstrup, H. Ibsen, I. Raymond, T. Bang-Hansen, C. Ellervik, E. Eriksen, L. Jensen, M. Jensen, M. Leth, A. Nygaard, J. Park, M. Schou, A. Therkelsen, J. Tilma, K. Vesterager.

*Finland*: P. Raatikainen, J. Airaksinen, O. Arola, J. Koistinen, H. Nappila, K. Peltomäki, V. Rasanen, T. Vasankari.

*France*: J.-Y. Le Heuzey, M. Galinier, Y. Gottwalles, F. Paganelli, P. Loiselet, J.-J. Muller, M.B. Koujan, A. Marquand, S. Destrac, O. Piot, N. Delarche, J.-P. Cebron, S. Boveda, M. Guenoun, D. Guedj-Meynier, D. Galley, J. Ohayon, S. Assouline, M. Zuber, P. Amarenco, E. Ellie, J. Kadouch, P.-Y. Fournier, J.-P. Huberman, M. Lemaire, G. Rodier, L. Milandre, X. Vandamme, I. Sibon, J.-P. Neau, M.H. Mahagne, A. Mielot, M. Bonnefoy, J.-B. Churet, V. Navarre, F. Sellem, G. Monniot, J.-P. Boyes, B. Doucet, M. Martelet, D. Obadia, B. Crousillat, J. Mouallem, E. Bearez, P. Nazeyrollas, J.P. Brugnaux, A. Fedorowsky, F. Casassus, J.-B. Berneau, F. Chemin, N. Falvo, J.-M. Perron, J.-E. Poulard, A. Barreau, C. Beltra, E. Corrihons, N. Decarsin, B. Dubois, E. Ducasse, X. Giry, A. Kemmel, S. Ledure, N. Lemaire, F. Robin, N. Rosolin, D. Sanchez, A. Suissa.

*Germany:* H. Darius, G. Königer, J. Purr, U. Gerbaulet, B.-T. Kellner, A. Kopf, T. Schäfer, H. Zauzig, P. Riegel, H. Hohensee, E. Eißfeller, W. Eder, G. Rehling, D. Glatzel, S. Zutz, G.-U. Heinz, H. Menke, A. Pustelnik, P. Sandow, N. Ludwig, H. Wiswedel, W. Wildenauer, C. Axthelm, T. Schwarz, A. Babyesiza, G. Stuchlik, H.-H. Zimny, M. Kropp, F. Kahl, A. Caspar, S. Omankowsky, T. Läßig, H.-J. Hartmann, G. Lehmann, H.-W. Bindig, G. Hergdt, D. Reimer, J. Hauk, W. Dorsch, J. Dshabrailov, H. Michel, K.-A. Rapp, R. Vormann, P. Mayer, U. Horstmeier, V. Eissing, H. Hey, H. Leuchtgens, V. Lilienweiß, K. Kolitsch, C. Schubert, H. Lauer, T. Buchner, G. Brauer, S. Kamin, K. Müller, M. Abdel-Qader, S. Baumbach, H.-H. Ebert, C. Schwencke, S. Schellong, P. Bernhardt, L. Karolyi, B. Sievers, W. Haverkamp, P. Salbach, J.-U. Röhnisch, S. Schoen, W. Erdle, T. Mueller, H. Mueller, V. Mitrovic, Z. Babjakova, K. Bergner, S. Boehme, K. Bonin, D. Buckert, F. Busch, U. Dichristin, S. Diez, A. Fleck, K. Flint, H. Floegel, C. Fritz, R. Frommhold, J. Gehre, J. Geyer, A. Grytzmann, M. Hahn, K. Helgert, K. Hubert, K. Kirchner-Volker, V. Klein, D. Kroll, A. Krueger, R. Lehmann, L. Mann, A. Maselli, G. Menken, K. Mikes, H. Mortan, N. Nasser, D. Nicolaus, A. Plauskat, L. Pomper, A. Quietzsch, C. Ravenhorst, C. Reichelt, C. Reimer, B. Schaefer, S. Scharrer, K. Schirmer, K. Schmidt, R. Schoene, J. Schulze, M. Schuppe, S. Simon, S. Sommer, K. Spranger, A. Talkenberger, K. Tauber, A. Tetlak, T. Toennishoff, R. Voelkel-Babyesiza, B. Voigts, U. Weiser, S. Wesendorf, S. Wildenauer, T. Wolf, J. Wurziger, J. Zak, H.-D. Zauzig, S. Ziefle, S. Zincke.

*Hungary:* M. Keltai, S. Vangel, G. Szalai, B. Merkely, S. Kancz, Z. Boda, A. Nagy, Z. Laszlo, A. Matoltsy, B. Gaszner, P. Polgar, T. Habon, E. Noori, G. Juhasz, N. Kanakaridisz, I. Szentpeteri, F. Juhasz, A. Vertes, A. Papp, Z. May, J. Ferenczi, M. Egyutt, E. Kis, G. Engelthaler, G. Szantai, E. Fulop, P. Gombos, D. Gulyas, P. Jen, E. Kiralyhazine Gyorke, M. Kovacs, S. Kovacsne Levang, S. Marianna, Z. Radics, N. Sydó, R. Szalo, A. Szilagyi, F. Sztanyik, B. Vandrus.

*Italy*: G. Agnelli, G. Ambrosio, E. Tiraferri, R. Santoro, S. Testa, G. Di Minno, M. Moia, T.M. Caimi, G. Martini, M. Tessitori, R. Cappelli, D. Poli, R. Quintavalla, F. Melone, F. Cosmi, A. Pizzini, G. Piseddu, R. Fanelli, C. Latella, R. Santi, L. Pancaldi, R. De Cristofaro, G. Palareti, A. De Blasio, J. Salerno Uriarte, F. Minetti, E.M. Pogliani, L.M. Lonati, M. Accogli, N. Ciampani, S. Malengo, M. Feola, A. Raisaro, L. Fattore, P. Grilli, F. Germini, M. Settimi, M. Alunni, G. Duranti, L. Tedeschi, G. Baglioni, G. Avanzino, M. Berardi, V. Pannacci, A. Giombolini, S. Nicoli, T. Scarponi, B. Allasia, P. Ricciarini, R. Nasorri, A. Argena, P. Bossolasco, P. Ronchini, A. Filippi, F. Tradati, C. Bulla, L. Donzelli, L. Foppa, M.L. Bottarelli, A. Tomasello, A. Mauric, C. Femiano, R. Reggio, F. Lillo, A. Mariani, F. Forcignanò, M. Volpe, M. D'Avino, M.G. Bongiorni, S. Severi, A. Capucci, C. Lodigiani, E. Salomone, G. Serviddio, C. Tondo, P. Golino, C. Mazzone, S. Iacopino, V. Pengo, M. Galvani, L. Moretti, P. Ambrosino, E. Banfi, V. Biagioli, A. Bianchi, G. Boggian, M. Breschi, S. Brusorio, F. Calcagnoli, G. Campagna, M. Carpenedo, C. Ciabatta, G. Ciliberti, G. Cimmino, C. D'Arienzo, L. Di Gennaro, M. Fedele, P.M. Ferrini, K. Granzow, G. Guazzaloca, F. Guerra, A. Lo Buglio, S. Longo, F. Macellari, E. Mesolella, E. Mollica Poeta, P. Occhilupo, V. Oriana, G. Rangel, L. Salomone, A. Scaccianoce, C. Scarone, L. Segreti, G. Sottilota, R. Villani, C. Zecca.

*Netherlands*: H. ten Cate, J.H. Ruiter, H. Klomps, M. Bongaerts, M.G.C. Pieterse, C. Guldener, J.-P. Herrman, G. Lochorn, A. Lucassen, H. Adriaansen, S.H.K. The, P.R. Nierop, P.A.M. Hoogslag, W. Hermans, B.E. Groenemeijer, W. Terpstra, C. Buiks, L.V.A. Boersma, M. Boersma-Slootweg, F. Bosman, M. Bosschaert, S. Bruin, I. Danse, J. De Graaf, J. de Graauw, M. Debordes, S. Dols, F. Geerlings, K. Gorrebeeck, A. Jerzewski, W. Jetten, M. Kelderman, T. Kloosterman, E.M. Koomen, J. Krikken, P. Melman, R. Mulder, A. Pronk, A. Stallinga-de Vos, J. te Kaat, P. Tonino, B. Uppelschoten, R. van de Loo, T. van der Kley, G. van Leeuwen, J.J. van Putten, L. Westerman.

*Norway:* D. Atar, E. Berge, P.A. Sirnes, E. Gjertsen, T. Hole, K. Erga, A. Hallaråker, G. Skjelvan, A. Østrem, B. Ghezai, A. Svilaas, P. Christersson, T. Øien, S. Høegh Henrichsen, J. Berg-Johansen, J.E. Otterstad, H. Antonsen, K. Ausen, H. Claussen, I. Dominguez, A. Jekthammer, A.B. Lensebraaten, V. Nilsen, M. O’Donovan, S. Rasmussen, K. Ringdalen, S. Strand.

*Poland:* J. Stepinska, R. Korzeniak, A. Gieroba, M. Biedrzycka, M. (Marcin) Ogorek, B. Wozakowska-Kaplon, K. Loboz-Grudzien, J. Kosior, W. Supinski, J. Kuzniar, R. Zaluska, J. Hiczkiewicz, L. Swiatkowska-Byczynska, L. Kucharski, M. Gruchala, P. Minc, M. Olszewski, G. Kania, M. Krzciuk, Z. Lajkowski, B. Ostrowska-Pomian, J. Lewczuk, E. Zinka, A. Karczmarczyk, M. Chmielnicka-Pruszczynska, M. Trusz-Gluza, G. Opolski, M. Bronisz, M. (Michal) Ogorek, G. Glanowska, P. Ruszkowski, K. Jaworska, R. Sciborski, B. Okopien, P. Kukla, I. Wozniak-Skowerska, K. Galbas, K. Cymerman, J. Jurowiecki, P. Miekus, W. Myszka, S. Mazur, R. Lysek, J. Baszak, T. Rusicka-Piekarz, G. Raczak, E. Domanska, J. Nessler, J. Lesnik, M. Ambicka, D. Andrzejewski, J. Araminowicz, A. Barszcz, R. Bartkowiak, J. Bartnik, M. Basiak, E. Bekieszczuk, L. Bernat, L. Biedrzycki, A. Biernacka, D. Blaszczyk, E. Broton, W. Brzozowski, M. Brzustowska, R. Bzymek, A. Chmielowski, P. Chojnowski, R. Cichomski, K. Cieslak, A. Cieszynska, B. Curyllo, M. Czamara, L. Danilowicz-Szymanowicz, B. Dolecka, L. Drelich, B. Dudzik-Richter, T. Dybala, M. Dziuba, W. Faron, M. Figura-Chmielewska, A. Frankiewicz, W. Gadzinski, E. Gasior, B. Gosciniecka, P. Gutknecht, M. Guziewicz, A. Jackun-Podlesna, G. Jaguszewska, J. Jankielewicz, A. Jaremczuk-Kaczmarczyk, M. Jargiello-Baszak, A. Jarzebowski, E. Jaskulska-Niedziela, M. Jaworska-Drozdowska, J. Kabat, A. Kaczmarzyk-Radka, K. Kalin, R. Kaliszczak, M. Kiliszek, M. Klata, M. Kluczewski, I. Kobielusz-Gembala, E. Kochanska, D. Kociolek, A. Kolodzinska, A. Komlo, A. Konopka, E. Korczowska, E. Kowal, H.K. Kowalczyk, E. Kremis, D. Kruczyk, A. Krzesiak-Lodyga, M. Krzyzanowski, W. Kurdzielewicz, D. Kustrzycka-Kratochwil, D. Lesniewska-Krynska, J. Leszczynski, E. Lewicka, E. Lichota, K. Lip, M. Loboz-Rudnicka, J. Luka, A. Lysek-Jozefowicz, M. Machnikowska, K. Majewska, R. Mariankowski, A. Markiewicz, M. Mazur, A. Metzgier-Gumiela, E. Miedlar, M. Mielcarek, J. Neubauer-Geryk, J. Niedek, A. Niemirycz-Makurat, A. Nowak, S. Nowak, B. Opielowska-Nowak, M. Ozgowicz, A. Pawelska-Buczen, E. Pawlik-Rak, R. Piotrowicz, P. Ptaszynski, A. Raczynska, W. Rogowski, J. Romanek, R. Romaszkiewicz, P. Rostoff, N. Roszczyk, D. Rozewska-Furmanek, J. Rychta, B. Rzyczkowska, A. Sidor, J. Skalska, M. Smichura, M. Splawski, P. Staneta, E. Staniszewska, J. Starak-Marciniak, M. Stopyra-Poczatek, M. Sukiennik-Kujawa, J. Szafranski, P. Szalecki, A. Szczepanska, W. Szkrobka, E. Szuchnik, A. Szulowska, G. Szumczyk-Muszytowska, M. Szwoch, T. Traczyk, M. Troszczynska, G. Trzcinski, S. Tybura, P. Walasik, M. Wegrzynowska, K. Wesolowska, W. Wieczorek, A. Wierzbicka, P. Wilczewski, M. Wilgat-Szecowka, P. Wojewoda, L. Wojnowski, M. Wrobel, K. Zakutynska-Kowalczyk, M. Zyczynska-Szmon.

*Russia*: E. Panchenko, V. Eltishcheva, R. Libis, S. Tereshchenko, S. Popov, G. Kamalov, D. Belenky, A. Zateyshchikova, E. Kropacheva, A. Kolesnikova, K. Nikolaev, L. Egorova, A. Khokhlov, E. Yakupov, M. Poltavskaya, D. Zateyshchikov, O. Drapkina, A. Vishnevsky, O. Barbarash, O. Miller, E. Aleksandrova, P. Chizhov, M. Sergeev, E. Shutemova, E. Mazur, K. Zrazhevskiy, T. Novikova, V. Kostenko, Y. Moiseeva, E. Polkanova, K. Sobolev, M. Rossovskaya, G. Zubeeva, Y. Shapovalova, O. Nagibovich, A. Edin, A. Agakhanyan, R. Batalov, Y. Belenkova, F. Bitakova, S. Chugunnaya, A. Dumikyan, S. Erofeeva, E. Gorbunova, T. Gorshkova, A. Gubanov, M. Gurmach, Y. Ivanova, T. Kolesova, D. Konyushenko, O. Korneeva, O. Kropova, P. Kuchuk, O. Kungurtseva, T. Kupriyanova, B. Kurylo, M. Kuvanova, O. Lebedeva, E. Lileeva, O. Machilskaya, T. Medvedeva, G. Monako, I. Motylev, G. Nagibovich, E. Novikova, Y. Orlov, Y. Osmolovskaya, A. Ovsannikova, D. Platonov, S. Rachkova, O. Sinitsina, S. Speshilova, O. Suslova, A. Ushakov, O. Volodicheva, O. Zemlianskaia, I. Zhirov, E. Zhuravleva, I. Zotova.

*Spain:* X. Viñolas, P. Alvarez Garcia, M.F. López Fernández, L. Tercedor, S. Tranche Iparraguirre, P. Torán Monserrat, E. Márquez Contreras, J. Isart Rafecas, J. Motero Carrasco, P. García Pavía, C. Gómez Pajuelo, C. Moro Serrano, L.F. Iglesias Alonso, A. Grande Ruiz, J. Mercé Klein, J.R. Gonzalez Juanatey, G. Barón Esquivias, I. Monte Collado, H. Palacín Piquero, C. Brotons Cuixart, M. Rodríguez Morató, J. Bayo I Llibre, C. Corros Vicente, M. Vida Gutierrez, F. Epelde Gonzalo, C.A. Almeida Fernández, N. Del Val Plana, E. Escrivá Montserrat, J.J. Montero Alía, M. Barreda González, M.A. Moleiro Oliva, J. Iglesias Sanmartín, M. Jiménez González, M. Rodriguez Álvarez, J. Herreros Melenchon, T. Ripoll Vera, F. Ridocci Soriano, L. Garcia Riesco, M.D. Marco Macian, J. Quiles Granado, M. Jimenez Navarro, J. Cosin Sales, J.V. Vaquer Perez, M. Vazquez Caamano, M.F. Arcocha Torres, G. Marcos Gomez, A. Iñiguez Romo, M.A. Prieto Diaz, C. (Carmela) Alonso, C. (Concepcion) Alonso, D. Alvarez, M. Alvarez, M. Amaro, N. Andere, J. Aracil Villar, R. Armitano Ochoa, A. Austria, S. Barbeira, E. Barraquer Feu, A. Bartes, V. Becerra Munoz, F.J. Bermudez Jimenez, A. Branjovich Tijuan, J. Cabeza Ramirez, M. Cabrera Ramos, E. Calvo Martinez, M. Campo Moreno, G. Cancho Corchado, M. Casanova Gil, M. Castillo Orive, D. Castro Fernandez, M. Cebollada del Misterio, R. Codinachs Alsina, A. Cortada Cabrera, J. Costa Pinto Prego de Faria, S. Costas, M.I. Cotilla Marco, M. Dachs, C.M. Diaz Lopez, A. Domenech Borras, A. Elorriaga Madariaga, A. Espallargas, M. Fernandez, E. Fernandez Escobar, E. Fernandez Mas, A. Ferrer, J. Fosch, M. Garcia Bermudez, V. Garcia Millan, M. Gavira Saenz, C. Gines Garcia, C. Gomez, Y. Gomez Perez, A. Gonzales Segovia, P. Gonzalez, L. Grigorian, A. Guerrero Molina, M. del C. Gutierrez del Val, B. Herrero Maeso, E. Hevia Rodriguez, A. Iglesias Garcia, M.J. Jimenez Fernandez, B. Jimeno Besa, P. Juan Salvadores, M.B. Lage Bouzamayor, I. Lasuncion, L.E. Lezcano Gort, M. Llobet Molina, M. Lopez, A. Manzanal Rey, J. Mara Guerra, S. Marcus, A. Martin Vila, M. Martinez Mena, P. Mazon, F. Mendez Zurita, G. Millán, M. Molina, P. Montero Alia, D. Montes, M. Moure Gonzalez, R.B. Munoz Munoz, A. Negrete Palma, H.N. Orellana Figueroa, V.M. Ortega, C. Ortiz Cortes, D. Otero Tomera, N. Palomo Merchan, I. Pareja Ibar, E. Pena Garcia, M. Pereda Armayor, M. Perez Carasa, I. Prieto, V. Quintern, R. Renom, L.M. Rincon Diaz, V. Rios, L. Riquelme Sola, R. Rivera, X. Robiro Robiro, M. Roca, C. Roca Saumell, C. Rodrigo, E. Rodriguez, M. Rodriguez Garcia, S. Saez Jimenez, P. Sanchez Calderon, L. Sanchez Mendez, S. Sanchez Parra, C. Santolaya, M.R. Senan Sanz, A. Seoane Blanco, E. Serralvo, N. Sierra, C. Simon Valero, J. Sorribes Lopez, M. Teixido Fontanillas, M. Terns Riera, G. Tobajas, C. Torres, J. Torres Marques, M. Ubeda Pastor.

*Sweden*: M. Rosenqvist, A. Wirdby, J. Linden, K. Henriksson, M. Elmersson, A. Egilsson, U. Börjesson, G. Svärd, B. Liu, A. Lindh, L.-B. Olsson, M. Gustavsson, L. (Lars) Andersson, L. (Lisbeth) Andersson, L. Benson, C. Bothin, A. Hajimirsadeghi, K. Kadir, M. Ericsson, A. Ohlsson, H. Lindvall, P. Svensson, K. Thorne, H. Handel, P. Platonov, B. Eriksson, I. Timberg, K. Romberg, M. Crisby, J.-E. Karlsson, S.A. Jensen, A. Andersson, L. Malmqvist, B. Martinsson, F. Bernsten, J. Engdahl, J. Thulin, A. Hot-Bjelac, P. Stalby, H. Aaröe, E. Ahbeck, H. Ahlmark, F. Al-Khalili, G. Bonkowski, S. Dzeletovic, A.-B. Ekstrand, G.-B. Eriksson, K. Floren, C. Grässjö, S. Hahn, P. Jaensson, B. Jansson, J.-H. Jansson, R.-M. Kangert, A. Koch, D. Kusiak, A. Lettenström, A. Lindberg, C.-J. Lindholm, A. Mannermyr, K. Mansson, M. Millborg, C. Nilsson, A.-M. Ohlin, A. Olofsson, A. Osberg, A. Pedersen, K. Risbecker, K. Rosenberg, J. Samuelsson, M. Shayesteh, K. Skoglund, M. Stjernberg, C. Thorsen.

*Switzerland*: J. Steffel, J.H. Beer, D. Shah, J. Debrunner, D. Amstutz, J. Bruegger, G. Elise, A. Grau, A. Guinand, I. Henriette, E. Saga, S. Winnik.

*Ukraine*: A. Parkhomenko, I. Rudyk, V. Tseluyko, O. Karpenko, S. Zhurba, I. Kraiz, I. Kupnovytska, N. Serediuk, Y. Mostovoy, O. Ushakov, O. Koval, I. Kovalskyi, Y. Svyshchenko, O. Sychov, M. Stanislavchuk, O. Kraydashenko, A. Yagensky, S. Tykhonova, B. Kurylo, I. Fushtey, R. Belegai, G. Berko, L. Burdeuna, O. Chabanna, I. Daniuk, A. Ivanov, E. Kamenska, P. Kaplan, O. Khyzhnyak, S. Kizim, O. Matova, O. Medentseva, V. Mochonyi, M. Mospan, V. Nemtsova, T. Ovdiienko, O. Palamarchuk, M. Pavelko, R. Petrovskyy, D. Plevak, O. Proshak, S. Pyvovar, L. Rasputina, O. Romanenko, O. Romanova, A. Sapatyi, O. Shumakov, R. Stets, L. Todoriuk, V. Varenov.

*UK:* D. Fitzmaurice, N. Chauhan, D. Goodwin, P. Saunders, R. Evans, J. Leese, P.S. Jhittay, A. Ross, M.S. Kainth, G. Pickavance, J. McDonnell, A. Williams, T. Gooding, H. Wagner, S. Suryani, A. Singal, S. Sircar, R. Bilas, P. Hutchinson, A. Wakeman, M. Stokes, N. Paul, M. Aziz, C. Ramesh, P. Wilson, S. Franklin, S. Fairhead, J. Thompson, V. St Joseph, G. Taylor, D. Tragen, D. Seamark, C. Paul, M. Richardson, A. Jefferies, H. Sharp, H. Jones, C. Giles, M. Page, O. Oginni, J. Aldegather, S. Wetherwell, W. Lumb, P. Evans, F. Scouller, N. Macey, Y. Stipp, R. West, S. Thurston, P. Wadeson, J. Matthews, P. Pandya, A. Gallagher, T. Railton, B. Sinha, D. Russell, J.A. Davies, P. Ainsworth, C.P. Jones, P. Weeks, J. Eden, D. Kernick, W. Murdoch, L. Lumley, R.P. Patel, S.W. Wong, M. Saigol, K. Ladha, K. Douglas, D.F. Cumberlidge, C. Bradshaw, G. Van Zon, K.P. Jones, M.J. Thomas, E. Watson, B. Sarai, N. Ahmad, W. Willcock, J. Cairns, S. Sathananthan, N. de Kare-Silver, A. Gilliland, E. Strieder, A. Howitt, B. Vishwanathan, N. Bird, D. Gray, P. Evans, M. Clark, J. Bisatt, J. Litchfield, E. Fisher, T. Fooks, A.R. Kelsall, E. Alborough, J. Wakeling, M. Parfitt, K. Milne, S. Rogers, R. Priyadharshan, J.L. Oliver, E. Davies, S. Abushal, M. Jacobs, C. Hutton, N.I. Walls, R. Thompson, C. Chigbo, S.M.A. Zaidi, M. Howard, K.C. Butter, S. Barrow, H. Little, I.U. Haq, L. Gibbons, S. Glencross, A.J. McLeod, K. Poland, C. Mulholland, A. Warke, P. Conn, G. Burns, R.N. Smith, S. Lowe, R. Kamath, H.S. Dau, J. Webster, I. Hodgins, S. Vercoe, P.C. Roome, H. Pinnock, J.R.A. Patel, A. Ali, N. Hart, R. Davies, E. Stuart, C.A. Neden, M. Danielsen, R. Heath, P. Sharma, S. Galloway, C. Hawkins, R. Oliver, M. Aylward, S. Mannion, M. Braddick, D. Edwards, A.C. Rothwell, A. Sabir, F. Choudhary, S. Khalaque, A. Wilson, S. Peters, W. Coulson, N. Roberts, A. Heer, S. Coates, B. Ward, D. Jackson, S. Walton, D. Shepherd, M. Sterry, T. Wong, M. Boon, R. Bunney, R. Haria-Shah, R.T. Baron, S. Davies, T. Schatzberger, N. Hargreaves, T. Stephenson, H. Choi, R. Batson, L. Lucraft, T. Myhill, S. Estifano, D. Geatch, J. Wilkinson, R. Veale, K. Forshaw, T. Davies, K. Zaman, P. Vinson, C. Liley, M. Bandrapalli, P. McGinty, R. Wastling, P. McEleny, A. Beattie, P. Cooke, M. Wong, J. Gunasegaram, M. Pugsley, S. Ahmad, C. A'Court, J. Ayers, J. Bennett, S. Cartwright, S. Dobson, C. Dooldeniya, A. Flynn, R. Fox, J. Goram, A. Halpin, A. Hay, P. Jacobs, L. Jeffers, L. Lomax, I. Munro, R. Muvva, M. Nadaph, K. Powell, S. Randfield, D. Redpath, R. Reed, M. Rickenbach, G. Rogers, P.B. Saunders, C. Seamark, J. Shewring, P. Simmons, H. Simper, H. Stoddart, A. Sword, N. Thomas, A. Thomson.

***Other countries***

*Australia:* H. Gibbs, A. Blenkhorn, B. Singh, W. Van Gaal, W. Abhayaratna, R. Lehman, P. Roberts-Thomson, J. Kilian, D. Coulshed, A. Catanchin, D. Colquhoun, H. Kiat, D. Eccleston, J. French, L. Zimmett, B. Ayres, T. Phan, P. Blombery, D. Crimmins, D. O’Donnell, A. Choi, P. Astridge, M. Arstall, N. Jepson, M. Binnekamp, A. Lee, J. Rogers, G. Starmer, P. Carroll, J. Faunt, A. Aggarwala, L. Barry, C. Batta, R. Beveridge, A. Black, M. Bonner, J. Boys, E. Buckley, M. Campo, L. Carlton, A. Connelly, B. Conway, D. Cresp, H. Dimitri, S. Dixon, M. Dolman, M. Duroux, M. Eskandari, R. Eslick, A. Ferreira-Jardim, T. Fetahovic, D. Fitzpatrick, R. Geraghty, J. Gibbs, T. Grabek, M. H Modi, K. Hayes, M.P. Hegde, L. Hesketh, B. Hoffmann, B. Jacobson, K. Johnson, C. Juergens, I. Kassam, V. Lawlor, M. Lehman, S. Lehman, D. Leung, S. Mackay, M. MacKenzie, C. McCarthy, C. McIntosh, L. McKeon, H. Morrison, C. Mussap, J.-D. Myers, V. Nagalingam, G. Oldfield, V. O’May, J. Palmer, L. Parsons, K. Patching, T. Patching, V. Paul, M. Plotz, S. Preston, H. Rashad, M. Ratcliffe, S. Raynes, J. Rose, L. Sanders, M. Seremetkoska, H. Setio, S. Shone, P. Shrestha, C. Singh, C. Singleton, N. Stoyanov, S. Sutcliffe, K. Swaraj, J. Tarrant, N. Thomas, S. Thompson, I.M. Tsay, M. Vorster, A. Waldman, L. Wallis, E. Wilford, K. Wong.

*Canada:* S.J. Connolly, A. Spyropoulos, J. Eikelboom, R. Luton, M. Gupta, A.S. Pandey, S. Cheung, R. Leader, P. Beaudry, F. Ayala-Paredes, J. Berlingieri, J. Heath, G. Poirier, M. Du Preez, R. Nadeau, G. Dresser, R. Dhillon, T. Hruczkowski, B. Schweitzer, B. Coutu, P. Angaran, P. MacDonald, S. Vizel, S. Fikry, R. Parkash, A. Lavoie, J. Cha, B. Ramjattan, J. Bonet, K. Ahmad, P. Angaran, L. Aro, T. Aves, K. Beaudry, C. Bergeron, C. Bergeron, J. Bigcanoe, N. Bignell, L. Breakwell, E. Burke, L. Carroll, B. Clarke, T. Cleveland, S. Daheb, P. Dehghani, I. Denis, Z. Djaidani, P. Dorian, S. Douglass, J. Dunnigan, A. Ewert, D. Farquhar, A. Fearon, L. Ferleyko, D. Fournier, B. Fox, M.-C. Grenier, W. Gulliver, K. Haveman, C. Hines, K. Hines, A.M. Jackson, C. Jean, G. Jethoo, R. Kahlon, S. Kelly, R. Kim, V. Korley, J. Kornder, L. Kwan, J. Largy, C. Lewis, S. Lewis, I. Mangat, R. Moor, J. Navratil, I. Neas, J. Otis, R. Otis, M. Pandey, F. Petrie, A. Pinter, M. Raines, P. Roberts, M. Robinson, G. Sas, S. Schulman, L. Snell, S. Spearson, J. Stevenson, T. Trahey, S. Wong, D. Wright.

*Egypt:* H. Ragy, A. Abd El-Aziz, S.K. Abou Seif, M.G. El Din, S. El Etriby, A. Elbahry, A. El-Etreby, M. Elkhadem, A. Katta, T. Khairy, A. Mowafy, M. Nawar, A. Ohanissian, A. Reda, M. Reda, H. Salem, N. Sami, S. Samir, M. Setiha, M. Sobhy, A. Soliman, N. Taha, M. Tawfik, E. Zaatout.

*South Africa:* B. Jacobson, D. Kettles, J. Bayat, H. Siebert, A. Horak, Y. Kelfkens, R. Garda, T. Pillay, M. Guerra, L. van Zyl, H. Theron, A. Murray, R. Louw, D. Greyling, P. Mntla, V. Ueckermann, R. Loghdey, S. Ismail, F. Ahmed, J. Engelbrecht, A. Ramdass, S. Maharajh, W. Oosthuysen, G. Angel, C. Bester, M. Booysen, C. Boshoff, C. Cannon, S. Cassimjee, C. Chami, G. Conway, A. Davids, L. de Meyer, G. Du Plessis, T. Ellis, L. Henley, M. Karsten, E. Loyd, J. Marks, L. Mavhusa, M. Mostert, A. Page, L. Rikhotso, M. Salie, J. Sasto, F. Shaik, A. Skein, L. Smith, G. Tarr, T. Tau, F. van Zyl.

*United Arab Emirates:* W. Al Mahmeed, G. Yousef, A. Agrawal, M. Nathani, M. Ibrahim, E.M. Esheiba, R. Singh, A. Naguib, M. Abu-Mahfouz, M. Al Omairi, A. Al Naeemi, R. Maruthanayagam, N. Bazargani, A. Wassef, R. Gupta, M. Khan, B. Subbaraman, A. Abdul, A. Al Mulla, S. El Bardisy, P. Haridas, S. Jadhav, K. Magdaluyo, M. Makdad, I. Maqsood, R. Mohamed, N. Sharma, R. Sharma, M. Thanzeel.

*USA:* S.Z. Goldhaber, R. Canosa, P. Rama, E. Blumberg, J. Garcia, P. Mullen, V. Wilson, A. Quick, K. Ferrick, W.M. Kutayli, M. Cox, M. Franco, S. Falkowski, R. Mendelson, M. Williams, S. Miller, S. Beach, N. Sharma, A. Alfieri, T. Gutowski, I. Haque, R. Reddy, W. Ahmed, P. Delafontaine, D. Diercks, D. Theodoro, K. Remmel, M. Alberts, R. Ison, H. Noveck, P. Duffy, S. Pitta, D. Nishijima, C. Treasure, N. Asafu-Adjaye, K. Ball, M. Bartlett, M. Bentley, S. Bowers, A. Brown, A. Browne, J. Cameron-Watts, M. Canova, D. Cassidy, K. Cervellione, S. Congal, J. DePauw, A. Dickerson, M. Eley, L. Evans, S. Felpel, K. Ferdinand, D. Fielder, P. Gentry, A. Haideri, F. Hakimi, T. Harbour, E. Hartranft, B. Hawkins, M. Headlee, L. Henson, C. Herrick, T. Hicks, S. Jasinski, K. Johnson, A. Jones, L. Jones, P. Jones, S. Karl, M. Keeling, J. Kerr, P. Knowles, J. Langdon, M. Lay, J.A. Lee, T. Lincoln, E. Malone, A. Merliss, D. Merritt, J. Minardo, B. Mooso, C. Orosco, V. Palumbo, M. Parker, T. Parrott, S. Paserchia, G. Pearl, J. Peterson, N. Pickelsimer, T. Purcell, J. Raynor, S. Raziano, C. Richard, T. Richardson, C. Robertson, A. Sage, T. Sanghera, P. Shaw, J. Shoemaker, K. Smith, B. Stephanie, A. Thatcher, H. Theobald, N. Thompson, L. Treasure, T. Tripti, C. Verdi, V. Worthy.
